# New Thiazolidine-4-One Derivatives as SARS-CoV-2 Main Protease Inhibitors

**DOI:** 10.3390/ph17050650

**Published:** 2024-05-17

**Authors:** Antonella Messore, Paolo Malune, Elisa Patacchini, Valentina Noemi Madia, Davide Ialongo, Merve Arpacioglu, Aurora Albano, Giuseppe Ruggieri, Francesco Saccoliti, Luigi Scipione, Enzo Tramontano, Serena Canton, Angela Corona, Sante Scognamiglio, Annalaura Paulis, Mustapha Suleiman, Helmi Mohammed Al-Maqtari, Fatma Mohamed A. Abid, Sarkar M. A. Kawsar, Murugesan Sankaranarayanan, Roberto Di Santo, Francesca Esposito, Roberta Costi

**Affiliations:** 1 Istituto Pasteur-Fondazione Cenci Bolognetti, Dipartimento di Chimica e Tecnologie del Farmaco, “Sapienza” Università di Roma, p.le Aldo Moro 5, 00185 Rome, Italy; antonella.messore@uniroma1.it (A.M.); elisa.patacchini@uniroma1.it (E.P.); davide.ialongo@uniroma1.it (D.I.); merve.arpacioglu@uniroma1.it (M.A.); aurora.albano@uniroma1.it (A.A.); giuseppe.ruggieri@uniroma1.it (G.R.); francesco.saccoliti90@gmail.com (F.S.); luigi.scipione@uniroma1.it (L.S.); roberto.disanto@uniroma1.it (R.D.S.); roberta.costi@uniroma1.it (R.C.); 2 Department of Life and Environmental Sciences, Faculty of Biology and Pharmacy, University of Cagliari, Cittadella Universitaria di Monserrato, ss554 Km 4500, 09045 Monserrato, Cagliari, Italy; paolo.malune@unica.it (P.M.); tramon@unica.it (E.T.); serena3canton@gmail.com (S.C.); angela.corona@unica.it (A.C.); sante.scognamiglio@unica.it (S.S.); annalaurapaulis@unica.it (A.P.); 3 Department of Chemistry, Sokoto State University, Sokoto 852101, Nigeria; masge007@gmail.com; 4 Department of Chemistry, College of Education, Hodeidah University, Hodeidah 207416, Yemen; helmi2007m@yahoo.com; 5 Department of Chemistry, Faculty of Science, Al-Azzaytuna University, Tarhuna 537622224, Libya; fatma_abeed@yahoo.com; 6 Laboratory of Carbohydrate and Nucleoside Chemistry, Department of Chemistry, University of Chittagong, Chittagong 4331, Bangladesh; akawsar@cu.ac.bd; 7 Medicinal Chemistry Research Laboratory, Birla Institute of Technology and Science Pilani, Pilani Campus, Pilani 333031, Rajasthan, India; murugesan@pilani.bits-pilani.ac.in

**Keywords:** SARS-CoV-2, COVID-19, main protease, thiazolidinone derivatives, small molecules, docking studies

## Abstract

It has been more than four years since the first report of SARS-CoV-2, and humankind has experienced a pandemic with an unprecedented impact. Moreover, the new variants have made the situation even worse. Among viral enzymes, the SARS-CoV-2 main protease (M^pro^) has been deemed a promising drug target vs. COVID-19. Indeed, M^pro^ is a pivotal enzyme for viral replication, and it is highly conserved within coronaviruses. It showed a high extent of conservation of the protease residues essential to the enzymatic activity, emphasizing its potential as a drug target to develop wide-spectrum antiviral agents effective not only vs. SARS-CoV-2 variants but also against other coronaviruses. Even though the FDA-approved drug nirmatrelvir, a M^pro^ inhibitor, has boosted the antiviral therapy for the treatment of COVID-19, the drug shows several drawbacks that hinder its clinical application. Herein, we report the synthesis of new thiazolidine-4-one derivatives endowed with inhibitory potencies in the micromolar range against SARS-CoV-2 M^pro^. In silico studies shed light on the key structural requirements responsible for binding to highly conserved enzymatic residues, showing that the thiazolidinone core acts as a mimetic of the Gln amino acid of the natural substrate and the central role of the nitro-substituted aromatic portion in establishing π-π stacking interactions with the catalytic His-41 residue.

## 1. Introduction

SARS-CoV-2 is the causative agent of the coronavirus disease 2019 (COVID-19) pandemic, a major public health threat that led to more than 774 million confirmed cases and over 7 million deaths as of January 2024 [1]. Despite the clinical perks of COVID-19 vaccines, this pandemic has persevered, with new variants constantly emerging [2]. Indeed, due to its rapid mutation rate and considerable immune evasion capabilities, SARS-CoV-2 continues to spread. Therefore, the ongoing transmission of SARS-CoV-2 underscores the need for the development of more potent antiviral agents, especially for noncritical patients, to prevent later hospitalization and death.

**Figure 1:**
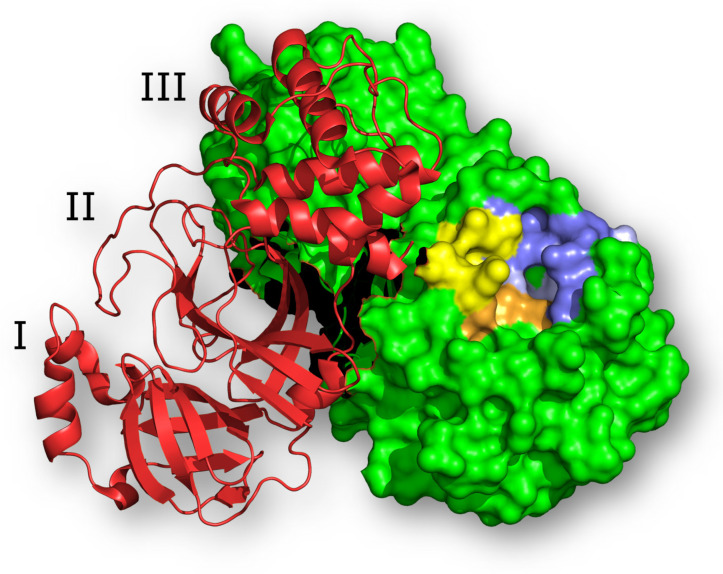
M^pro^ of SARS-CoV-2 in its dimeric form. One monomer is shown as a red cartoon with details of domains I, II, and III. The second monomer is shown in green surfaces with details of subsite S1 (in yellow), S1’ (in orange), S2 (in light blue), and S3 (in blue).

Over four years have passed since the first case of a new coronavirus infection (SARS-CoV-2) in the city of Wuhan (Hubei, China). Since then, a significant number of experimental and clinical studies have been conducted to identify effective approaches to its prevention and treatment [3]. Great successes have been achieved in the identification of potential viral targets. Among the various genes and proteins encoded by SARS-CoV2, the main protease (M^pro^) has emerged as the most studied nonstructural protein and “druggable” target for drug development against SARS-CoV-2 [4]. This is a cysteine protease that is involved in the production of 16 nonstructural proteins (nsps) from the polyproteins pp1a and pp1ab, which are produced through translation of ORF1a and ORF1b. In detail, M^pro^ is a homodimer, whereby each protomer exhibits three-domain structures, an N-finger and chymotrypsin-like domain I, domain II, and domain III (Figure 1) [5,6]. Between domains I and II is located a substrate-binding site, which is a substrate-binding pocket, consisting of the previously identified and characterized subsites S1, S2, S4, and S1’ [6,7].

M^pro^ plays an indispensable role in a viral life cycle, and its inhibition impedes the formation of replication-essential enzymes, thus blocking viral replication. Furthermore, considering that this protein has no human homolog [8] and a high interspecific similarity among coronaviruses [9], it is evident that M^pro^ is one of the most promising drug targets. Moreover, the extreme degree of conservation of the M^pro^ observed in analyzing over 13.7 million SARS-CoV-2 variants’ sequences reinforces the role of M^pro^ as an appealing target for antiviral drugs, exhibiting broad-spectrum efficacy against all variants of SARS-CoV-2 [10].

Up until now, various SARS-CoV-2 M^pro^ inhibitors have been reported in the literature. These can be divided into two classes: class (i) non-covalent inhibitors, which primarily act as orthosteric and allosteric inhibitors, and class (ii) inhibitors, which covalently bind the M^pro^ catalytic pocket and are characterized as either peptidomimetic or non-peptidomimetic inhibitors.

Class (i) includes many inhibitors characterized by different chemical structures [11,12,13,14]. However, all these inhibitors defect due to suboptimal potency, toxicity, imperfect pharmacokinetic (PK) properties, or drug resistance issues [15].

**Figure 2:**
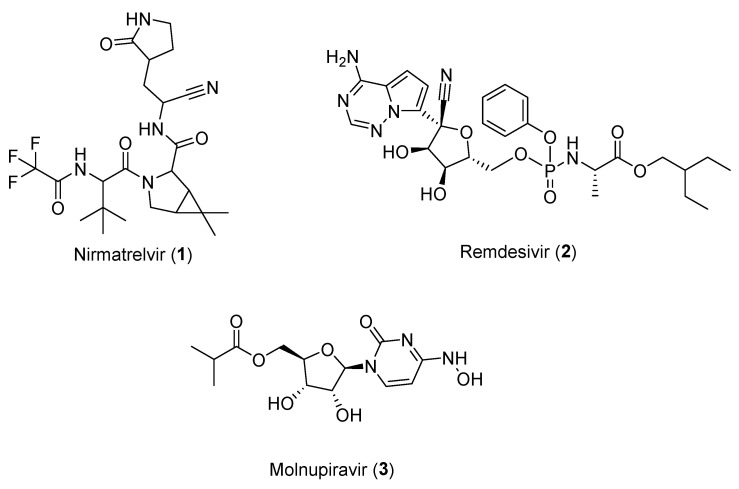
FDA-approved antiviral drugs **1**–**3**.

Among class (ii) inhibitors, only nirmatrelvir (**1**, Figure 2) (Paxlovid, ritonavir-boosted) achieved the approval of the Food and Drug Administration (FDA) as the first oral drug to treat mild to moderate COVID-19 in adults who are at high risk of severe COVID-19 [16].

Despite the importance of the availability of an approved drug, three major issues cannot be overlooked. First, studies have shown efficacy only if the administration occurs within 5 days of the symptom onset [17]. Second, one significant drawback of nirmatrelvir-ritonavir is the notable potential risk for drug interactions, primarily stemming from ritonavir’s inhibition of the cytochrome P450 3A4 enzyme. Indeed, co-administration with ritonavir is strictly necessary because it inhibits the cytochrome that is most responsible for nirmatrelvir metabolism. However, even if this inhibition leads to the heightened pharmacodynamic activity of nirmatrelvir, it also leads to several drug–drug interactions with commonly used drugs that are also metabolized by the same cytochrome [18,19]. Last, it should not be forgotten that drug-resistant variants against currently approved drugs, including nirmatrelvir, have already emerged [15,20]. Hence, the development of next-generation antivirals is imperative. Indeed, even if the FDA has approved two other drugs up to today (the remdesivir (**2**) and the molnupiravir (**3**), two viral polymerase inhibitors, Figure 2), several drawbacks hinder their effectiveness and their use in clinical practice (i.e., weak activity in SARS-CoV-2 infected individuals, the intravenous route of administration of **2** that limits its use to hospital settings, the unfavorable benefit/risk ratio, etc.) [21].

Recently, thiazolidine-4-ones moiety showed inhibitory activity with IC_50_ values within the μM range against SARS-CoV-2 M^pro^ [22]. This is a scaffold that has long been known in medicinal chemistry for its different pharmacological activities. Indeed, thiazolidinones are intriguing heterocyclic five-membered moieties present in a diverse array of natural bioactive compounds and drugs [23]. This moiety possesses diverse biological applications, and it has been known to display antimicrobial [24,25,26], antifungal [27], antitubercular [28,29], anticancer [30], anticonvulsant [31], anti-inflammatory [32], antiviral, and anti-HIV activities [33]. Therefore, it is not surprising that this moiety has also shown activity against SARS-CoV-2. In particular, Petrou et al. reported a series of thiazolidine-4-one derivatives as SARS-CoV-2 M^pro^ inhibitors [22]. M^pro^ is known for cleaving its substrate after Gln, which is preceded by Leu and before a Ser or Ala or Gly amino acid (Leu-Gln ↓ Ser/Ala/Gly). The presence of a thiazolidinone scaffold appears to mimic the role of Gln amino acid found in the natural substrate. This moiety could potentially be positioned within the S1 subsite in the active center of the enzyme, where Gln is naturally placed, highlighting the central role of this scaffold in the M^pro^ inhibition. However, only a few derivatives showed M^pro^ inhibitory activity up to 50 μM, even though all the tested compounds proved to be cytotoxic against human lung fibroblast MRC-5 cells, negatively affecting the cell viability by more than 50% at the concentrations equal to their IC_50_ values. Hence, deepening structure–activity relationships (SARs) within this promising class of molecules are attractive.

**Figure 3:**
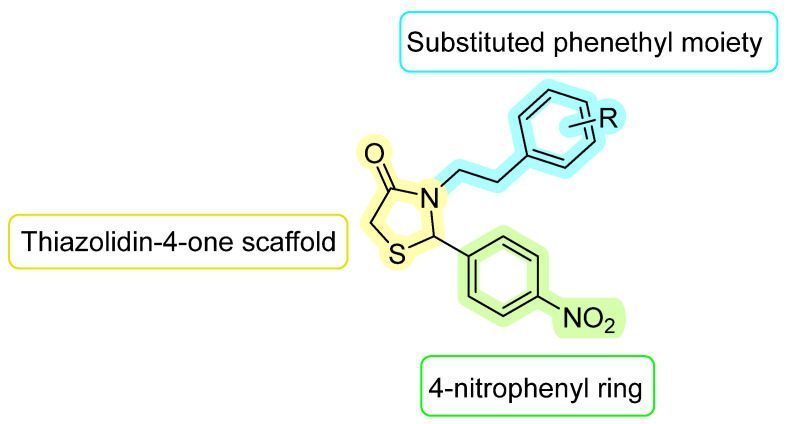
Newly designed thiazolidine-4-one derivatives **4a**–**i**.

In light of our long-standing expertise in the design and synthesis of small molecules endowed with antiviral and antiretroviral activities [34,35,36], together with our recent findings about the development of new anti-SARS-CoV-2 agents [37], we designed and synthesized a new series of thiazolidin-4-ones derivatives (**4a**–**i**) as SARS-CoV-2 M^pro^ inhibitors to expand the SARs within this class, characterized by a variously substituted phenethyl moiety and a 4-nitrophenyl ring linked to the thiazolidine-4-one scaffold (Figure 3). The newly designed compounds were conceived taking into account literature data describing that the presence of halophenyl rings like chlorophenyl or fluorophenyl substituents on the thiazolidin-4-one scaffold increases the antiviral activity [33,38]. Furthermore, we applied a molecular docking protocol to predict the possible binding mode of the compounds on SARS-CoV-2 M^pro^, shedding light on the main structural features involved in enzymatic inhibition.

## 2. Results

### 2.1. Chemistry

**Scheme 1:**
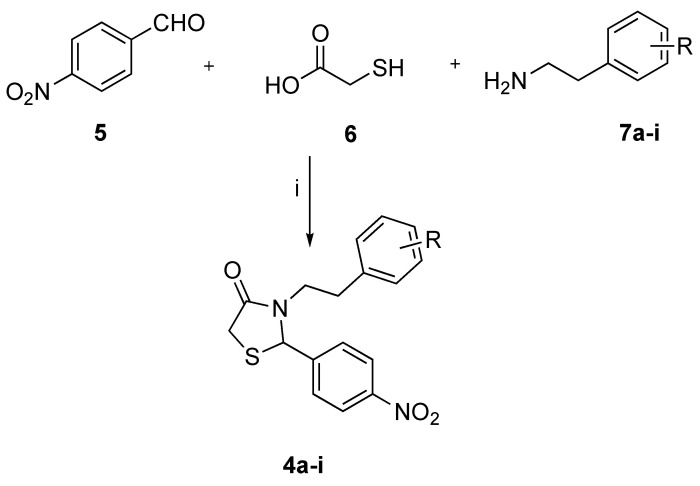
Synthetic route to **4a**–**i** derivatives: *i*: toluene dry, reflux, 48 h, 70–87% yield.

The synthesis of the new thiazolidine-4-ones compounds was achieved by a multicomponent one-pot reaction, with the proper phenethylamine and aldehyde in the presence of mercaptoacetic acid, in refluxing toluene, as previously reported [39,40]. In particular, the appropriate Schiff base/azomethine intermediates, obtained by reacting the commercially available 4-nitrobenzaldehyde with an equimolar amount of the properly substituted phenethylamine derivatives (as detailed in Materials and Methods), underwent a cyclocondensation reaction with an excess amount of α-mercaptoalkanoic acids such as a mercaptoacetic acid. The reactions were carried out in refluxing *dry* toluene (the use of a *dry* solvent was chosen to improve the yields given that the cyclization reaction is concomitant with elimination of water) for 48 h (Scheme 1), affording the desired compounds **4a**–**i**. Noteworthy, compound **4a** was obtained as previously described [41]. The final products were obtained as pure solids in satisfactory to good yields, and all of them were characterized by elemental analysis and spectroscopic methods (FTIR, ^1^H NMR, and ^13^C NMR).

The detailed structures of newly synthesized compounds **4a**–**i** are listed in Table 1.

### 2.2. Biological Activities

All the newly synthesized compounds were tested for their inhibitory properties against SARS-CoV-2 M^pro^ in a biochemical assay, using GC376 as positive control (Table 2).

While some of the compounds showed inactivity (IC_50_ > 30 μM, which is the highest concentration tested), approximately half of them demonstrated the ability to inhibit SARS-CoV-2 M^pro^ with IC_50_ values in the micromolar range. In detail, among the halogenated derivatives, the presence of a fluorine atom in the para position on the phenethyl moiety (**4d**) or of a chlorine atom in the ortho/meta position (**4g** and **4h**, respectively) seemed to improve the inhibitory activity. On the other hand, the introduction of these atoms in positions different from the above-mentioned seemed to be detrimental to the activity, suggesting that the fine-tuning of the phenethyl substitution pattern was relevant to the enzymatic inhibition. Likewise, the introduction of a methoxy substituent on the phenethyl moiety led to inactive compounds (**4e** and **4f**), just like the unsubstituted counterpart **4a**. Although some compounds have been shown to inhibit Mpro SARS-CoV-2 in biochemical assays, all compounds of this chemical serie were inactive when tested on the SARS-CoV-2 viral replication in cell culture, despite not showing toxicity (Table 2).

### 2.3. Binding Modes Prediction

All the compounds were subjected to a docking protocol in order to predict their binding modes, to gather insights about the role of the halogen atom on the phenethyl moiety, and to rationalize the effect of its substitution pattern on the activity.

**Figure 4:**
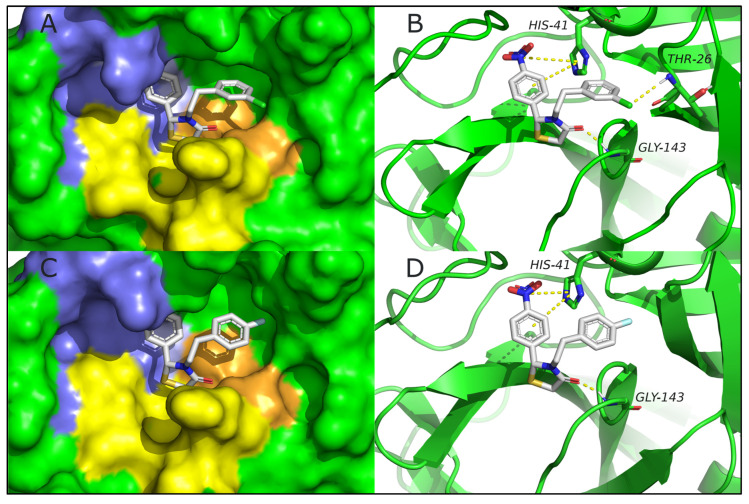
Binding of **4h** (**A**,**B**) and **4d** (**C**,**D**). Protein is shown in green; **4h** and **4d** are shown in white. (**A**,**C**) The protein surface is shown with details of subsite S1 (in yellow), S1’ (in orange), S2 (in light blue), and S3 (in blue). (**B**,**D**) A focus on the residues involved in the protein–ligand interaction.

Subsites have previously been described within the active site based on interactions with peptide-based inhibitors [6,7] and are depicted in Figure 4A. In agreement with the results of the in vitro assays, the best active compound **4h** also shows the best binding pose: the chlorine-substituted derivative is the only one capable of establishing an additional bond. In particular, this type of atom is halogen-bonded with the hydrogen of the residue Thr-26, as shown in Figure 4B. The substitution in the meta position is the best one to favor this type of bond, which, however, is also established for the ortho-substituted derivative **4g**. In the para-substituted derivative **4i**, on the other hand, the excessive steric hindrance completely prevents the formation of this interaction. This hypothesis is also supported by the higher activity of the para-fluorine-substituted counterpart **4d** (Figure 4C,D). Indeed, having the fluorine atom with a smaller atomic radius than chlorine, compound **4d** might be able to better fit in the lipophilic pocket near to the subsite S1’, resulting in a better activity of **4d**. In addition to the peculiar halogen bond, **4h** shows interactions in common with all derivatives. In detail, the carbonyl group of the thiazolidinone core is hydrogen-bonded with the Gly-143, and the nitro-substituted aromatic portion establishes π–π stacking and cation–π interaction with His-41 that is part of the catalytic dyad (consistent with the SARS chymotrypsin-like protease [42]) of the enzyme.

To obtain a more precise ranking of the ligand docking poses, we rescored all the ligand–protein complexes using the MM-GBSA approach implemented in Schrödinger’s Maestro suite [43]. This method was used to calculate the ligand binding free energy to proteins. It exploits molecular dynamics simulations with an explicit solvent of the protein–ligand complex to obtain a series of snapshots for which energies are computed. This change in the solvation method not only requires reweighting of energies with implicit solvent energies, which is not typically performed, but also requires flexibility on the part of the protein, enhancing the accuracy of binding energy calculations [44]. The results are summarized in Table 3. As expected, the best activity of **4h** is also reflected by the scoring values obtained by MM-GBSA protocol. This compound was predicted to be the most favored to bind the protein, in terms of binding energy (kcal/mol). On the other hand, the worst results are shown by **4c**, **4e**, **4f**, **4i**, which are inactive according to the biological results.

## 3. Materials and Methods

### 3.1. Chemistry

#### 3.1.1. General Instrumentation

Melting points were determined on a Leica Galen III micro melting point apparatus and were uncorrected. IR spectra were recorded on an FTIR spectroscopy (FTIR Spectrometer Frontier, Perkin Elmer, Waltham, MA, USA) model, using a Perkin Elmer instrument, to determine the vibration of the binding compounds in the wavenumber range of 4000 cm^−1^ and 600 cm^−1^. The ^1^H NMR (400 MHz) and ^13^C NMR (100 MHz) were recorded on Bruker Avance II 400 MHz NMR spectrometer using deuterated chloroform (CDCl_3_, Sigma-Aldrich, Merck, Darmstadt, Germany, 99.96%), acetone (CD_3_COCD_3_, Merck, Darmstadt, Germany, 99.9%), or dimethyl sulfoxide (DMSO-d_6_, Sigma-Aldrich, 99.9%) as the solvent. Tetramethylsilane (TMS) was used as an internal standard to report chemical changes in parts per million (δ ppm). Abbreviations for peak patterns are as follows: s (singlet), d (doublet), dd (double doublet), t (triplet), m (multiplet), and q (quadruplet). Hertz values were used for coupling constants. Analytical TLC was performed using Merck’s pre-coated silica gel plates 60 F254, with spots visualized using UV light (254 nm). For column chromatography, silica gel (230–400 mesh) was used as the stationary phase, and hexane-ethyl acetate as the mobile phase. The starting materials used in the research were commercially available. Aromatic aldehyde (4-nitrobenzaldehyde), the mercaptoacetic acid, and the amine (2-phenylethylamine and its derivatives) were purchased from Sigma-Aldrich, while the others were purchased from Qrec, such as *dry* toluene, ethyl acetate (EtOAc), hydrochloric acid (HCl), sodium hydrogen carbonate (NaHCO_3_), magnesium sulfate (MgSO_4_), and absolute ethanol (EtOH). The concentration of solutions after reactions and extractions involved the use of a rotary evaporator (Büchi, Flawil, Switzerland) operating at a reduced pressure (ca. 20 Torr). Organic solutions were dried over anhydrous magnesium sulfate. Melting point (°C), yield (%), chromatographic system, recrystallization solvent, IR, ^1^H NMR, and ^13^C NMR were reported.

#### 3.1.2. General Experimental Procedures

**General Procedure for the synthesis of thiazolidine-4-one derivatives 4a**–**i**. To a stirred mixture of 4-nitrobenzaldehyde (1.0 mmol, 0.15 g) in *dry* toluene (30 mL), mercaptoacetic acid (2.0 mmol, 0.18 g) was added under reflux conditions, followed by the addition of the properly substituted phenethylamine (1.0 mmol). The mixture was refluxed (115–120 °C) for 48 h until the complete consumption of the phenethylamine. After that, the solvent was evaporated under reduced pressure and the residue was dissolved in ethyl acetate. The organic layer was washed with 5% aqueous HCl, water, 5% aqueous NaHCO_3_, and brine. The organic layer was dried over magnesium sulfate and the solvent was evaporated under reduced pressure to yield a crude product that was purified by column chromatography using hexane–ethyl acetate as a solvent system. For each derivative, the amount of the starting material (proper phenethylamine), yield (%), melting point, recrystallization solvent, IR, ^1^H-NMR, and ^13^C NMR were reported (see Supplementary Materials for the proper IR, ^1^H-NMR, and ^13^C NMR spectrum).

#### 3.1.3. Specific Procedures and Characterization

**2-(4-nitrophenyl)-3-(phenylethyl)-1,3-thiazolidin-4-one (4a)**. Compound **4a** (previously reported in ref. [41]) was prepared from 2-phenylethylamine (0.001 mol, 0.16 g) using the general procedure above; pale yellow amorphous solid; yield: 85%. M.p 125 °C; ethanol; IR (cm^−1^): 2928 (C-H sp^3^), 1667 (C=O), 1597 and 1436 (C=C), 1340 (N=O), 1021 (C-N); ^1^H NMR (400 MHz, CDCl_3_): δ 2.75–2.94 (3H, m, H-6 and H-7), δ 3.72 (1H, d, *J* = 15.6 Hz, H-5a), δ 3.82 (1H, d, *J* = 15.6 Hz, H-5b), δ 3.98–4.04 (1H, m, H-6 or H-7), δ 5.30 (1H, s, H-2), δ 7.13 (2H, d, *J* = 6.4, H-Ar), δ 7.29–7.34 (5H, m, H-Ar), δ 8.23 (2H, d, *J* = 8.8 Hz, H-Ar); ^13^C NMR (100 MHz, CDCl_3_) δ 32.7 (CH_2_*C*H_2_-Ar), 33.6 (SCH_2_), 45.0 (*C*H_2_CH_2_-Ar), 63.0 (SCHN), 124.5 (C3 and C5 ArNO_2_), 127.1 (C4 Ar), 127.9 (C2 and C6 ArNO_2_), 128.9, 129.0 (C2, C3, C5 and C6 Ar), 138.3 (C1 Ar), 146.9 and 148.4 (C1 and C4 ArNO_2_), 171.3 (CO); Anal. calcd for C_17_H_16_N_2_O_3_S: C, 62.18; H, 4.91; N, 8.53; S, 9.76%. Found: C, 61.98; H, 4.89; N, 8.51; S, 9.75.

**2-(4-nitrophenyl)-3-(2-fluorophenylethyl)-1,3-thiazolidin-4-one (4b)**. Compound **4b** was prepared from 2-fluorophenylethylamine (0.001 mol, 0.14 g) using the general procedure above; yellow crystals; yield: 75%. M.p 150–151 °C; ethanol; IR (cm^−1^): 2929 (C-H sp^3^), 1667 (C=O), 1507 and 1445 (C=C), 1339 (N=O), 1221 (C-F), 1023 (C-N); ^1^H NMR (400 MHz, CDCl_3_): δ 2.78–3.00 (3H, m, H-6 and H-7), δ 3.69 (1H, d, *J* = 15.6 Hz, H-5a), δ 3.79 (1H, d, *J* = 15.6 Hz, H-5b), δ 3.92–4.01 (1H, m, H-6 or H-7), δ 5.41 (1H, s, H-2), δ 6.98–7.32 (4H, m, H-Ar), δ 7.34 (2H, d, *J* = 8.0, H-Ar), δ 8.19 (2H, d, *J* = 8.0 Hz, H-Ar); ^13^C NMR (100 MHz, CDCl_3_) δ 26.9 (CH_2_*C*H_2_-Ar), 32.6 (SCH_2_), 43.3 (*C*H_2_CH_2_-Ar), 62.8 (SCHN), 115.6 (C3 ArF), 124.4 (C3 and C5 ArNO_2_), 125.1 (C1 ArF), 128.0 (C2 and C6 ArNO_2_), 128.9 (C4 ArF), 131.1 (C6 ArF), 146.7 (C5 ArF), 148.3 (C1 and C4 ArNO_2_), 159.9 and 162.4 (C2 ArF), 171.1 (CO); Anal. calcd for C_17_H_15_FN_2_O_3_S: C, 58.95; H, 4.37; F, 5.48; N, 8.09; S, 9.26. Found: C, 58.93; H, 4.36; F, 5.47; N, 8.07; S, 9.24.

**2-(4-nitrophenyl)-3-(3-fluorophenylethyl)-1,3-thiazolidin-4-one (4c)**. Compound **4c** was prepared from 3-fluorophenylethylamine (0.001 mol, 0.14 g) using the general procedure above; light-orange crystals; yield: 78%. M.p 165–167 °C; ethanol; IR (cm^−1^): 2932 (C-H sp^3^), 1665 (C=O), 1517 and 1434 (C=C), 1342 (N=O), 1247 (C-F), 1016 (C-N); ^1^H NMR (400 MHz, CDCl_3_): δ 2.79–2.89 (3H, m, H-6 and H-7), δ 3.68 (1H, d, *J* = 15.6 Hz, H-5a), δ 3.78 (1H, d, *J* = 15.6 Hz, H-5b), δ 3.81–3.89 (1H, m, H-6 or H-7), δ 5.29 (1H, s, H-2), δ 6.78–7.13 (3H, m, H-Ar), δ 7.21 (1H, s, H-Ar), δ 7.30 (2H, d, *J* = 7.6 Hz, H-Ar), δ 8.29 (2H, d, *J* = 7.6 Hz, H-Ar); ^13^C NMR (100 MHz, CDCl_3_) δ 32.5 (CH_2_*C*H_2_-Ar), 33.1 (SCH_2_), 44.6 (*C*H_2_CH_2_-Ar), 62.8 (SCHN), 113.8 (C4 ArF), 115.5 (C2 ArF), 124.3 (C6 ArF and C3 and C5 ArNO_2_), 127.8 (C2 and C6 ArNO_2_), 130.3 (C5 ArF), 140.6 (C1 ArF), 146.7 and 148.3 (C1 and C4 ArNO_2_), 161.7 and 164.1 (C3 ArF), 171.2 (CO); Anal. calcd for C_17_H_15_FN_2_O_3_S: C, 58.95; H, 4.37; F, 5.48; N, 8.09; S, 9.26. Found: C, 58.98; H, 4.38; F, 5.49; N, 8.11; S, 9.28.

**2-(4-nitrophenyl)- 3-(4-fluorophenylethyl)-1,3-thiazolidin-4-one (4d)**. Compound **4d** was prepared from 4-fluorophenylethylamine (0.001 mol, 0.14 g) using the general procedure above; orange crystals; yield: 80%. M.p 149–151 °C; ethanol; IR (cm^−1^): 2932 (C-H sp^3^), 1667 (C=O), 1516 and 1416 (C=C), 1342 (N=O), 1222 (C-F), 1014 (C-N); ^1^H NMR (400 MHz, CDCl_3_): δ 2.76–2.86 (3H, m, H-6 and H-7), δ 3.78 (1H, d, *J* = 15.6 Hz, H-5a), δ 3.88 (1H, d, *J* = 15.6 Hz, H-5b), δ 3.93–4.01 (1H, m, H-6 or H-7), δ 5.31 (1H, s, H-2), δ 6.91 (2H, d, *J* = 7.6, H-Ar), δ 7.17 (2H, d, *J* = 7.6, H-Ar), δ 7.32 (2H, d, *J* = 8.0, H-Ar), δ 8.20 (2H, d, *J* = 8.0 Hz, H-Ar); ^13^C NMR (100 MHz, CDCl_3_) δ 32.6 (CH_2_*C*H_2_-Ar and SCH_2_), 44.9 (*C*H_2_CH_2_-Ar), 62.8 (SCHN), 115.6 (C3 and C5 ArF), 124.5 (C3 and C5 ArNO_2_), 127.7 (C2 and C6 ArNO_2_), 130.1 (C2 and C6 ArF), 133.7 (C1 ArF), 148.8 and 148.3 (C1 and C4 ArNO_2_), 160.6 and 163.04 (C4 ArF), 171.2 (CO); Anal. calcd for C_17_H_15_FN_2_O_3_S: C, 58.95; H, 4.37; F, 5.48; N, 8.09; S, 9.26. Found: C, 58.92; H, 4.36; F, 5.47; N, 8.10; S, 9.25.

**2-(4-nitrophenyl)-3-(2-chlorophenylethyl)-1,3-thiazolidin-4-one (4e)**. Compound **4e** was prepared from 2-chlorophenylethylamine (0.001 mol, 0.16 g) using the general procedure above; pale-yellow solid; yield 70%. M.p 123–124.5 °C; ethanol; IR (cm^−1^): 3059 (C-H sp^2^), 2939 (C-H sp^3^), 1667 (C=O), 1441 and 1516 (C=C aromatic), 1338 (NO_2_), 737 (C-Cl); ^1^H NMR (400 MHz, CDCl_3_) δ: 2.86–3.10 (3H, m, H-6 and H-7), δ 3.75 (1H, d, *J *= 16.0 Hz, H-5a), δ 3.84 (1H, d, *J *= 16.0 Hz, H-5b), δ 3.87–3.95 (1H, m, H-6 or H-7), δ 5.39 (1H, s, H-2), δ 7.23 (2H, d, *J *= 8.0 Hz, H-Ar), δ 7.33–7.39 (5H, m, H-Ar), δ 8.24 (2H, d, *J *= 8.0 Hz, H-Ar); ^13^C NMR (100 MHz, CDCl_3_) δ 31.1 (CH_2_*C*H_2_-Ar), 32.7 (SCH_2_), 43.0 (*C*H_2_CH_2_-Ar), 63.0 (SCHN), 124.4 (C3 and C5 ArNO_2_), 127.3 (C5 ArCl), 128.0 (C2 and C6 ArNO_2_), 128.6 (C4 ArCl), 129.7 (C6 ArCl), 131.1 (C3 ArCl), 133.9 (C2 ArCl), 135.8 (C1 ArCl), 146.7 and 148.3 (C1 and C4 ArNO_2_), 171.1 (CO); Anal. calcd for C_17_H_15_ClN_2_O_3_S: C, 56.28; H, 4.17; Cl, 9.77; N, 7.72; S, 8.84. Found: C, 58.25; H, 4.16; Cl, 9.75; N, 7.70; O, S, 8.82.

**2-(4-nitrophenyl)-3-(3-chlorophenylethyl)-1,3-thiazolidin-4-one (4f)**. Compound **4f** was prepared from 3-chlorophenylethylamine (0.001 mol, 0.16 g) using the general procedure above; dark-yellow solid; yield: 76%. M.p 140–142 °C; ethanol; IR (cm^−1^): 3020 (C-H sp^2^), 2956 (C-H sp^3^), 1675 (C=O), 1452 and 1579 (C=C aromatic), 1345 (NO_2_), 744 (C-Cl); ^1^H NMR (400 MHz, DMSO) δ: 2.88–3.04 (2H, m, H-8a,b), δ 3.45 (2H, dd, *J *= 6.2 Hz, H-2), δ 3.69 (2H, m, H-7), δ 6.02 (1H, s, H-4), δ 7.03 (1H, dd, *J *= 7.4 Hz, H-Ar), δ 7.11 (5H, q, *J *= 1.17 Hz, H-Ar), δ 8.25 (2H, d, *J *= 8.0 Hz, H-Ar); ^13^C NMR (100 MHz, CDCl_3_) δ 32.6 (CH_2_*C*H_2_-Ar), 33.1 (SCH_2_), 44.6 (*C*H_2_CH_2_-Ar), 62.9 (SCHN), 124.5 (C3 and C5 ArNO_2_), 126.9 (C4 ArCl), 127.2 (C6 ArCl), 127.8 (C2 and C6 ArNO_2_), 128.8 (C2 ArCl), 130.1 (C5 ArCl), 134.6 (C3 ArCl), 140.1 (C1 ArCl), 146.6 and 148.3 (C1 and C4 ArNO_2_), 171.1 (CO); Anal. calcd for C_17_H_15_ClN_2_O_3_S: C, 56.28; H, 4.17; Cl, 9.77; N, 7.72; S, 8.84%. Found: C, 58.31; H, 4.18; Cl, 9.79; N, 7.74; S, 8.86.

**2-(4-nitrophenyl)-3-(4-chlorophenylethyl)-1,3-thiazolidin-4-one (4g)**. Compound **4g** was prepared from 4-chlorophenylethylamine (0.001 mol, 0.16 g) using the general procedure above; dark-yellow solid; yield: 81%. M.p 129–131 °C; ethanol; IR (cm^−1^): 3015 (C-H sp^2^), 2940 (C-H sp^3^), 1635 (C=O), 1467 and 1518 (C=C aromatic), 1372 (NO_2_), 735 (C-Cl); ^1^H NMR (400 MHz, DMSO) δ: 2.90–3.00 (2H, m, H-8a,b), δ 3.60 (2H, dd, *J* = 6.2 Hz, H-2), δ 3.67 (2H, m, H-7a,b), δ 6.02 (1H, s, H-4), δ 7.17 (2H, m, H-Ar), δ 7.32 (2H, m, H-Ar), δ 7.54 (2H, m, H-Ar), δ 8.16 (2H, m, H-Ar); ^13^C NMR (100 MHz, CDCl_3_) δ 32.6 (CH_2_*C*H_2_-Ar), 32.7 (SCH_2_), 44.7 (*C*H_2_CH_2_-Ar), 62.8 (SCHN), 124.5 (C3 and C5 ArNO_2_), 127.7 (C2 and C6 ArNO_2_) 128.9 (C3 and C5 ArCl), 130.0 (C2 and C6 ArCl), 132.8 (C4 ArCl), 136.5 (C1 ArCl), 146.7 and 148.3 (C1 and C4 ArNO_2_), 171.2 (CO); Anal. calcd for C_17_H_15_ClN_2_O_3_S: C, 56.28; H, 4.17; Cl, 9.77; N, 7.72; O, 13.23; S, 8.84. Found: C, 58.32; H, 4.16; Cl, 9.76; N, 7.71; S, 8.85.

**2-(4-nitrophenyl)-3-(2-methoxyphenylethyl)-1,3-thiazolidin-4-one (4h)**. Compound **4h** was prepared from 2-methoxyphenylethylamine (0.001 mol, 0.15 g) using the general procedure above; yellowish pale crystals; yield: 81%. M.p 150–151.5 °C; ethanol; IR (cm^−1^): 2930 (C-H), 1237 (C-O-C), 1674 (C=O), 1337 (NO_2_); ^1^H NMR (400 MHz, CDCl_3_) δ 2.78–2.89 (3H, m, H-6 and H-7), δ 3.72 (3H, s, OCH_3_), δ 3.72 (1H, d, *J *= 15.2, H-5a), δ 3.81 (1H, d, *J *= 15.2, H-5b), δ 3.95–4.2 (1H, m, H-6 or H-7), δ 5.37 (1H, s, H-2), δ 6.84 (1H, d, *J *= 8.0, H-Ar), δ 6.91–6.94 (1H, m, H-Ar), δ 7.12 (1H, d, *J *= 7.2, H-Ar), δ 7.25–7.33 (4H, m, H-Ar), δ 8.22 (1H, d, *J *= 8.8, H-Ar); ^13^C NMR (100 MHz, CDCl_3_) δ 28.3 (CH_2_*C*H_2_-Ar), 32.7 (SCH_2_), 43.0 (*C*H_2_CH_2_-Ar), 55.1 (SCHN), 62.8 (OCH_3_), 110.3 (C3 ArOCH_3_), 120.8 (C5 ArOCH_3_), 124.2 (C3 and C5 ArNO_2_), 126.5 (C4 ArOCH_3_), 128.0 (C2 and C6 ArNO_2_), 128.3 (C1 ArOCH_3_), 130.6 (C6 ArOCH_3_), 147.0 and 148.5 (C1 and C4 ArNO_2_), 157.4 (C2 ArOCH_3_), 171.1 (CO); Anal. calcd for C_18_H_18_N_2_O_4_S: C, 60.32; H, 5.06; N, 7.82; S, 8.95. Found: C, 60.35; H, 5.07; N, 7.84; S, 8.97.

**2-(4-nitrophenyl)-3-(4-methoxyphenylethyl)-1,3-thiazolidin-4-one (4i)**. Compound **4i** was prepared from 4-methoxy phenylethylamine (0.001 mol, 0.15 g) using the general procedure above; dark-yellow crystals; yield: 87%. M.p 158–159.5 °C; ethanol; IR (cm^−1^): 2932 (C-H), C=O (1665), 1240 (C-O-C), 1514 and 1406 (C=C aromatic), 1340 (NO_2_); ^1^HNMR (400 MHz, CDCl_3_) δ 2.71–2.82 (3H, m, H-6 and H-7), δ 3.70–4.08 (3H, d, *J *= 15.2, H-5a, H-5b and H-6 or H-7a), δ 3.82 (3H, s, OCH_3_), δ 5.31 (1H, s, H-2), δ 6.85 (2H, d, *J *= 7.2, H-Ar), δ 7.04 (2H, d, *J *= 7.2, H-Ar), δ 7.34 (2H, d, *J *= 7.6, H-Ar), δ 8.23 (2H, d, *J *= 7.6, H-Ar); ^13^C NMR (100 MHz, CDCl_3_) δ 32.5 (CH_2_*C*H_2_-Ar), 32.6 (SCH_2_), 45.0 (*C*H_2_CH_2_-Ar), 55.3 (SCHN), 62.9 (OCH_3_), 114.2 (C3 and C5 ArOCH_3_), 124.4 (C3 and C5 ArNO_2_), 127.8 (C2 and C6 ArNO_2_), 129.6 (C2 and C6 ArOCH_3_), 130.1 (C1 ArOCH_3_), 146.8 and 148.2 (C1 and C4 ArNO_2_), 158.6 (C1 ArOCH_3_), 171.1 (CO); Anal. calcd for C_18_H_18_N_2_O_4_S: C, 60.32; H, 5.06; N, 7.82; O, 17.86; S, 8.95. Found: C, 60.29; H, 5.05; N, 7.81; S, 8.94.

### 3.2. SARS-CoV-2 Mpro Biochemical Assay

The inhibition of SARS-CoV-2 M^pro^ was measured using a FRET assay, as previously described. Briefly, SARSCoV-2 M^pro^ was expressed and purified as described in Biolatti et al. [45]; it was pre-incubated with different concentrations of the compounds at 37 °C for 30 min, in a mixture containing 20 mM Tris−HCl pH 7.3, 100 mM NaCl, 1 mM EDTA, 5 mM TCEP, and 0.1% BSA [10]. Subsequently, 12 μM of FRET substrate (peptide DABCYL-KTSAVLQ↓SGFRKM-EDANS) was added and the reaction was carried out at room temperature for 15 min, after which the fluorescent signal (ex/em 320/480) was acquired using a plate reader (PerkinElmer, Waltham, MA, USA) [46].

### 3.3. SARS-CoV-2 Viral Replication Assay in Vero-E6 GFP

SARS-CoV-2 replication assay was performed as described [47]. Vero-E6 GFP cells were maintained in DMEM (Gibco, Thermo Fisher Scientific, Waltham, MA, USA) supplemented with 10% *v*/*v* FBS (Gibco), 0.075% Na bicarbonate (7.5% solution, Gibco), and 1x Pen-strep (Euroclone, Pero, Milan, Italy). On the first day, cells were seeded at 104 cells/well in 96-well black plates (PerkinElmer). The following day, cells were incubated, with or without compounds, in the presence of 2 μM P-gp inhibitor CP-100356 [48], at different concentrations, and infected with SARS-CoV-2 BetaCoV/Belgium/GHB-03021/2020 strain (kindly provided by KU Leuven, amplified in Vero-E6-GFP) with a multiplicity of infection (MOI) of 0.01. Compound GC376 was used as positive control, in the presence of 2 μM P-gp inhibitor CP-100356 [47]. The media was removed 72 h post-infection, and the total well GFP fluorescence was measured using Victor 3 with 485/535 nm excitation wavelength. All SARS-CoV-2-related work was carried out in a certified, high-containment biosafety level-3 facility at the University of Cagliari. The inhibition of viral replication was calculated as a percentage of virus-induced cytopathic effect on infected untreated controls. EC_50_ value was calculated using Prism 9. Version 9.1.2 via non-linear regression.

### 3.4. Cell Viability Effect Measured in Vero-E6 GFP

Vero-E6 GFP cells were seeded at 10^4^ cells/well in 96-well black plates (PerkinElmer). The following day, cells were incubated, with or without compounds, with 2 μM P-gp inhibitor CP-100356 [47]. The media was removed 24 h after treatment, and the total well GFP fluorescence was measured using Victor NIVO 5 with 485/535 nm excitation wavelength. The cytotoxic effect of compounds was calculated as a percentage of cell viability with respect to untreated control. Data were analyzed using Prism 9. Version 9.1.2.

### 3.5. Docking Procedures

Docking calculations were conducted for all the compounds employing the Glide tool implemented in Maestro [43]. Considering the variety of crystallographic structures available, it was opted to use the three lowest resolution proteins from SARS-CoV-2 in a monomeric form, without mutations and excluding the covalent inhibitors (PDB codes 7JKV, 7D1M, and 8OKN) [49,50]. Before employing these receptor structures in docking calculations, preparation steps were undertaken using the Protein Preparation Wizard utility within the Maestro software package. The receptor structures were preprocessed by assigning bond orders, adding hydrogens, and generating physiological pH states using the EPIK tool. Subsequently, the “Minimize and Delete Waters” tool was utilized to minimize the overall protein structures, with restrained heavy atoms and the removal of all water molecules. To prepare the ligands for docking calculations, a separate tool within the Schrodinger software suite known as “LigPrep” was utilized. Specifically, all the hydrogen atoms were added, and the appropriate ionization states were calculated. The docking grid boxes were centered based on the respective co-crystal ligands with grid box dimensions of 20 Å × 20 Å × 20 Å for all the models. Finally, docking runs were executed using the standard Glide protocol with a rigid treatment of the protein, employing standard settings [51,52]. The best-scoring complexes in terms of GlideScore were selected and then subjected to MM-GBSA analysis to enhance the accuracy of binding energy calculations compared with molecular docking energies. MM-GBSA calculations were performed allowing a protein flexibility of up to 5 Å, utilizing the OPLS_2005 force field with the VSGB 2.0 solvation model [53]. Images were rendered using Pymol [54].

## 4. Conclusions

In the fight against COVID-19 and other emerging and re-emerging pathogenic viruses, the development of small molecules inhibiting highly conserved and attractive viral targets is mandatory. In this context, the SARS-CoV-2 M^pro^ enzyme plays a key role thanks to its pivotal role in the viral lifecycle and a high interspecific similarity among coronaviruses. Even though some reports of small molecule inhibitors targeting M^pro^ have been described in the literature and the FDA-approved drug nirmatrelvir is currently used in therapy, several drawbacks that cannot be overlooked prompt researchers to find new effective M^pro^ inhibitors. Here, we report the synthesis of a set of new thiazolidine-4-one derivatives endowed with inhibitory activities against SARS-CoV-2 M^pro^ in the low micromolar range. Noteworthy, the thiazolidinone core seems to act as a mimetic of the Gln amino acid of the natural substrate. Docking studies allowed us to rationalize the structural features involved in the interaction within the enzyme, highlighting their binding close to highly conserved amino acidic residues. Moreover, our in silico studies showed the central role of the nitro-substituted aromatic portion that establishes π–π stacking and cation–π interaction with the catalytic His-41 residue, providing valuable insights to guide the medicinal chemistry research to develop new SARS-CoV-2 M^pro^ inhibitors, possibly endowed with a broad spectrum of activity.

## Figures and Tables

**Table 1:** Detailed chemical structures of the newly synthesized compounds **4a**–**i**.

Compound	R	Yield (%)
**4a**	H	85
**4b**	*o*-F	75
**4c**	*m*-F	78
**4d**	*p*-F	80
**4e**	*o*-OCH_3_	70
**4f**	*p*-OCH_3_	76
**4g**	*o*-Cl	81
**4h**	*m*-Cl	81
**4i**	*p*-Cl	87

**Table 2:** Effect of compounds **4a**–**i** on SARS-CoV-2 Mpro activity and on SARS-CoV-2 viral replication.

Compound	SARS-CoV-2 M^pro a^ IC_50_ (μM)	^c^ EC_50_ + CP_100356	^d^ CC_50_ +CP_100356
**4a**	>30 (53%) ^b^	>100	>100
**4b**	>30 (70%)	>100	>100
**4c**	>30 (87%)	>100	>100
**4d**	27.5 ± 5.5	>100	>100
**4e**	>30 (54%)	>100	>100
**4f**	>30 (82%)	>100	>100
**4g**	26 ± 4	>100	>100
**4h**	18.5 ± 2.5	>100	>100
**4i**	>30 (54%)	>100	>100
**GC376**	0.00014 ± 0.00001	0.626 ± 0.06	>100

^a^ Compound concentration required to reduce the SARS-2 M^pro^ activity by 50%. ^b^ Percentage of control activity measured in the presence of the indicated drug concentration. ^c^ Compound concentration required to reduce the viral replication by 50%. ^d^ Compound concentration required to reduce Vero E6-GFP viability by 50%. IC_50_ was expressed as a means ± standard deviation of three independent experiments.

**Table 3:** Estimated free binding energies of tested compounds according to MM-GBSA calculations.

Compound	R	MM-BGSA ΔG Binding
**4a**	H	−54.73
**4b**	*o*-F	−59.22
**4c**	*m*-F	−42.11
**4d**	*p*-F	−63.40
**4e**	*o*-CH_3_	−45.62
**4f**	*p*-CH_3_	−48.79
**4g**	*o*-Cl	−64.06
**4h**	*m*-Cl	−69.85
**4i**	*p*-Cl	−47.11

## Data Availability

Data are contained within the article and Supplementary Materials.

## Supplementary Materials

The following are available online at https://www.mdpi.com/article/10.3390/ph17050650/s1. Figure S1: FTIR Spectrum for **4a**; Figure S2: ^1^H NMR Spectrum for **4a**; Figure S3: ^13^C NMR Spectrum for **4a**; Figure S4: FTIR Spectrum for **4b**; Figure S5: ^1^H NMR Spectrum for **4b**; Figure S6: ^13^C NMR Spectrum for **4b**; Figure S7: FTIR Spectrum for **4c**; Figure S8: ^1^H NMR Spectrum for **4c**; Figure S9: ^13^C NMR Spectrum for **4c**; Figure S10: FTIR Spectrum for **4d**; Figure S11: ^1^H NMR Spectrum for **4d**; Figure S12: ^13^C NMR Spectrum for **4d**; Figure S13: FTIR Spectrum for **4e**; Figure S14: ^1^H NMR Spectrum for **4e**; Figure S15: ^13^C NMR Spectrum for **4e**; Figure S16: FTIR Spectrum for **4f**; Figure S17: ^1^H NMR Spectrum for **4f**; Figure S18: ^13^C NMR Spectrum for **4f**; Figure S19: FTIR Spectrum for **4g**; Figure S20: ^1^H NMR Spectrum for **4g**; Figure S21: ^13^C NMR Spectrum for **4g**; Figure S22: FTIR Spectrum for **4h**; Figure S23: ^1^H NMR Spectrum for **4h**; Figure S24: ^13^C NMR Spectrum for **4h**; Figure S25: FTIR Spectrum for **4i**; Figure S26: ^1^H NMR Spectrum for **4i**; Figure S27: ^13^C NMR Spectrum for **4i**.
