# Supplementary material for: New Thiazolidine-4-One Derivatives as SARS-CoV-2 Main Protease Inhibitors
Source: Pharmaceuticals (Basel). 2024 May 17;17(5):650. doi: 10.3390/ph17050650 (PMC11124136; doi:10.3390/ph17050650)
Supplement: Supplementary file 1 [file pharmaceuticals-17-00650-s001.zip › pharmaceuticals-2989896-supplementary.pdf]

New thiazolidine-4-one derivatives as SARS-CoV-2 main protease inhibitors

Antonella Messore [1,†], Paolo Malune [2], Elisa Patacchini [1,†], Valentina Noemi Madia [1,*], Davide Ialongo [1], Merve Arpacioglu [1], Aurora Albano [1], Giuseppe Ruggieri [1], Francesco Saccoliti [1], Luigi Scipione [1], Enzo Tramontano [2], Serena Canton [2], Angela Corona [2], Sante Scognamiglio [2], Annalaura Paulis [2], Mustapha Suleiman [3], Helmi Mohammed Al-Maqtari [4], Fatma Mohamed A. Abid [5], Sarkar M.A. Kawsar [6], Murugesan Sankaranarayanan [7], Roberto Di Santo [1], Francesca Esposito [2,*] and Roberta Costi [1]

[1] Istituto Pasteur-Fondazione Cenci Bolognetti, Dipartimento di Chimica e Tecnologie del Farmaco, "Sapienza" Università di Roma, p.le Aldo Moro 5, I-00185 Rome, Italy; antonella.messore@uniroma1.it (A.M.); elisa.patacchini@uniroma1.it (E.P.); valentinanoemi.madia@uniroma1.it (V.N.M.); davide.ialongo@uniroma1.it (D.I.); merve.arpacioglu@uniroma1.it (M.A.); aurora.albano@uniroma1.it (A.A.); giuseppe.ruggieri@uniroma1.it (G.R.); francesco.saccoliti90@gmail.com (F.S.); luigi.scipione@uniroma1.it (L.S.); roberto.disanto@uniroma1.it (R.D.S.); roberta.costi@uniroma1.it (R.C.).

[2] Department of Life and Environmental Sciences, Faculty of Biology and Pharmacy, University of Cagliari, Cittadella Universitaria di Monserrato, ss554 Km 4500, 09045 Monserrato, Cagliari, Italy; paolo.malune@unica.it; (P.M.); tramon@unica.it (E.T.); serena3canton@gmail.com (S.C.); sante.scognamiglio@unica.it (S.S.); angela.corona@unica.it (A.C.); annalaurapaulis@unica.it (A.P.); francescaesposito@unica.it (F.E.).

[3] Department of Chemistry, Sokoto State University, Sokoto, 852101, Nigeria; masge007@gmail.com (Mus.S.)

[4] Department of Chemistry, College of Education, Hodeidah University, Hodeidah, 207416, Yemen; helmi2007m@yahoo.com (H.M.A.M.)

[5] Department of Chemistry, Faculty of Science, Al-Azzaytuna University, Tarhuna, 537622224, Libya; fatma_abeed@yahoo.com (F.M.A.A.)

[6] Laboratory of Carbohydrate and Nucleoside Chemistry, Department of Chemistry, University of Chittagong, Chittagong 4331, Bangladesh; akawsar@cu.ac.bd (S.M.A.K.)

[7] Medicinal Chemistry Research Laboratory, Birla Institute of Technology and Science Pilani, Pilani Campus, Pilani 333031, Rajasthan, India; murugesan@pilani.bits-pilani.ac.in (Mur.S.)

† These authors contributed equally to this work.

* Correspondence: valentinanoemi.madia@uniroma1.it, Tel.: +39-06-4991-3965 (V.N.M.); francescaesposito@unica.it, Tel.: +39- 070-6754533 (F.E.).

Contents

Figure S1: FTIR Spectrum for compound **4a**;

Figure S2: ^{1}H NMR Spectrum for compound **4a**;

Figure S3: ^{13}C NMR Spectrum for compound **4a**;

Figure S4: FTIR Spectrum for compound **4b**;

Figure S5: ^{1}H NMR Spectrum for compound **4b**

Figure S6: ^{13}C NMR Spectrum for compound **4b**;

Figure S7: FTIR Spectrum for compound **4c**;

Figure S8: ^{1}H NMR Spectrum for compound **4c;**

Figure S9: ^{13}C NMR Spectrum for compound **4c;**

Figure S10: FTIR Spectrum for compound **4d**;

Figure S11: ^{1}H NMR Spectrum for compound **4d;**

Figure S12: ^{13}C NMR Spectrum for compound **4d;**

Figure S13: FTIR Spectrum for compound **4e**;

Figure S14: ^{1}H NMR Spectrum for compound **4e;**

Figure S15: ^{13}C NMR Spectrum for compound **4e;**

Figure S16: FTIR Spectrum for compound **4f**;

Figure S17: ^{1}H NMR Spectrum for compound **4f;**

Figure S18: ^{13}C NMR Spectrum for compound **4f;**

Figure S19: FTIR Spectrum for compound **4g**;

Figure S20: ^{1}H NMR Spectrum for compound **4g;**

Figure S21: ^{13}C NMR Spectrum for compound **4g;**

Figure S22: FTIR Spectrum for compound **4h**;

Figure S23: ^{1}H NMR Spectrum for compound **4h;**

Figure S24: ^{13}C NMR Spectrum for compound **4h;**

Figure S25: FTIR Spectrum for compound **4i**;

Figure S26: ^{1}H NMR Spectrum for compound **4i;**

Figure S27: ^{13}C NMR Spectrum for compound **4i;**

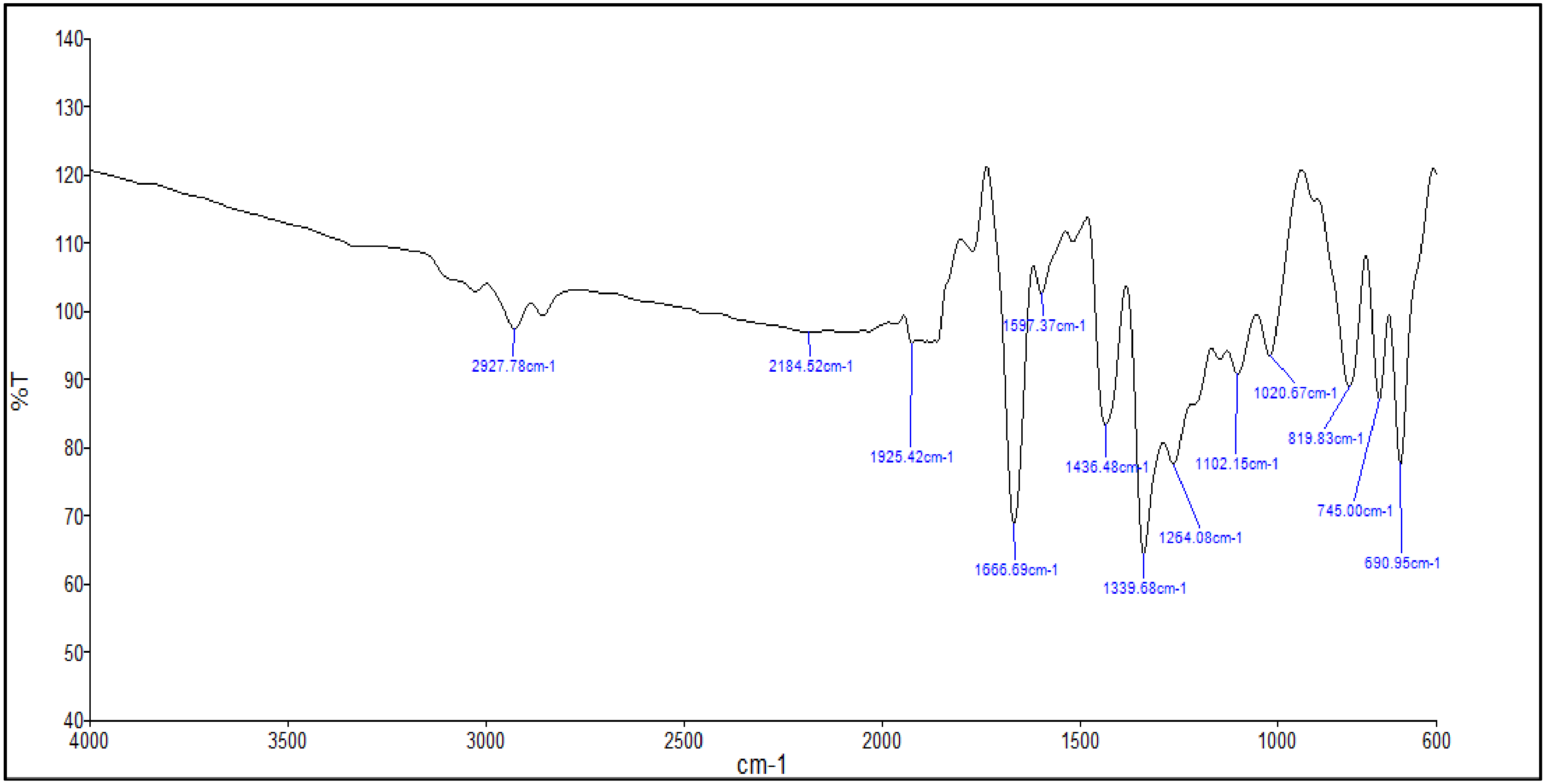

Figure S1. FTIR Spectrum for compound **4a**

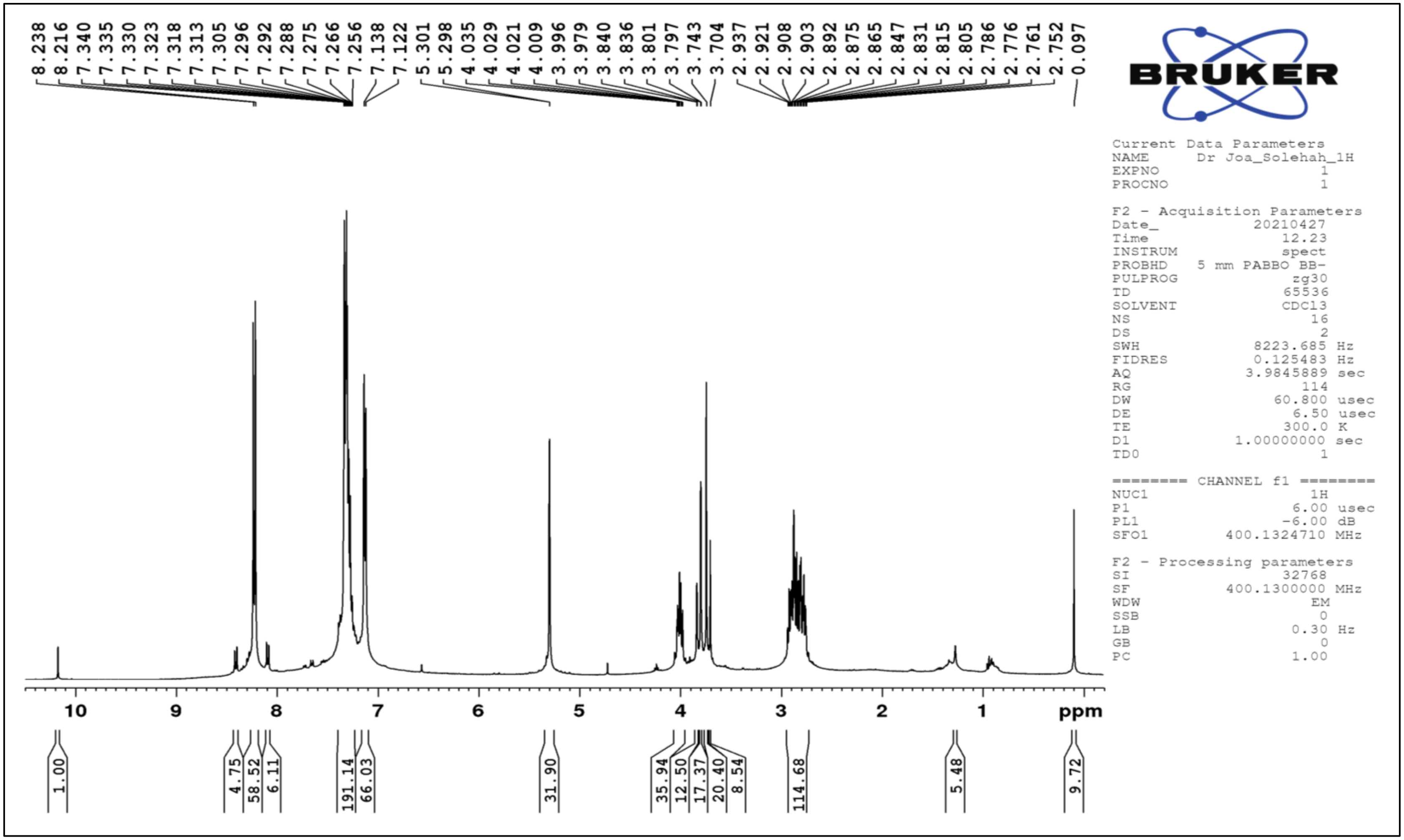

Figure S2. ^{1}H NMR Spectrum for compound **4a**

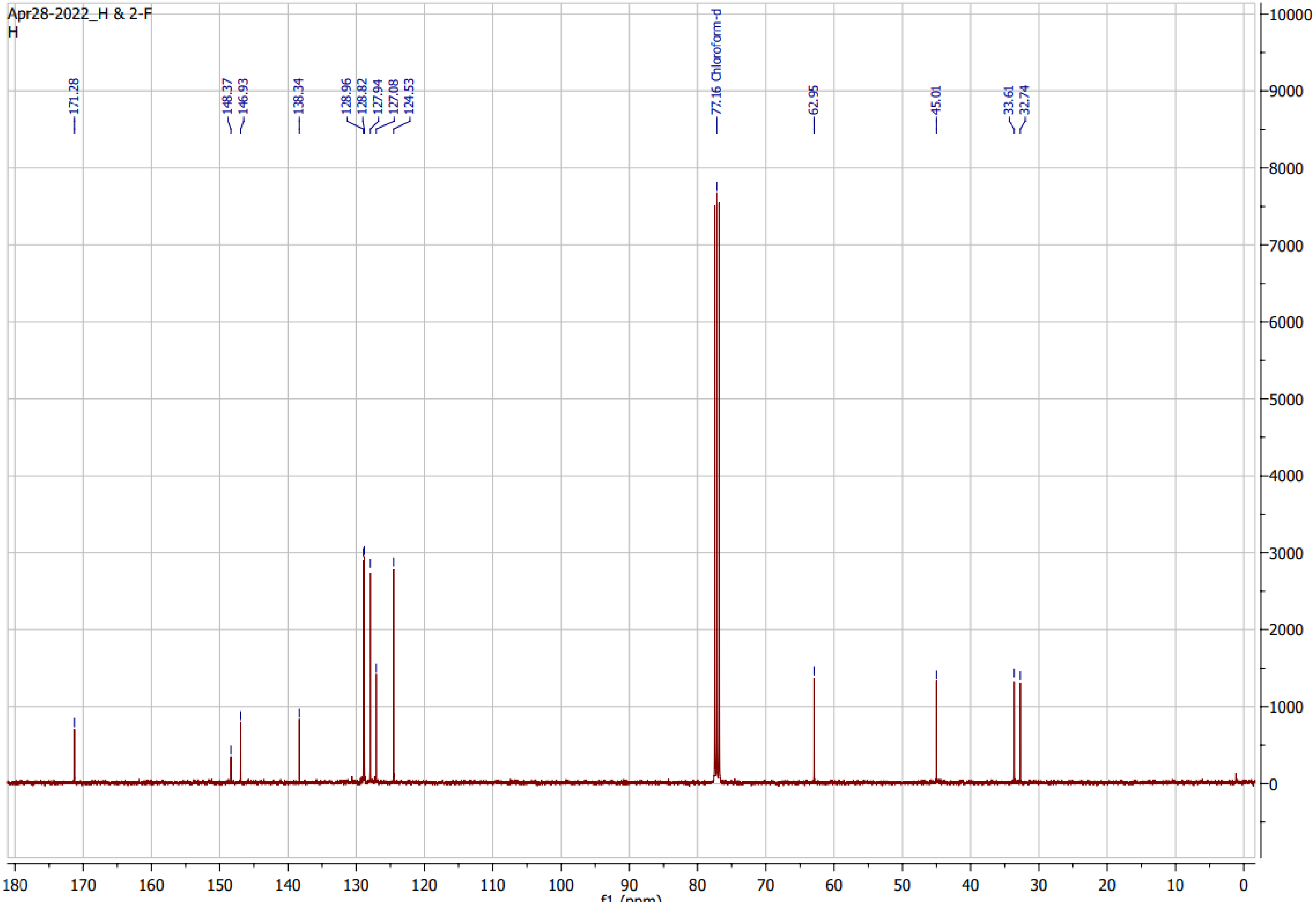

Figure S3. ^{13}C NMR Spectrum for compound **4a**

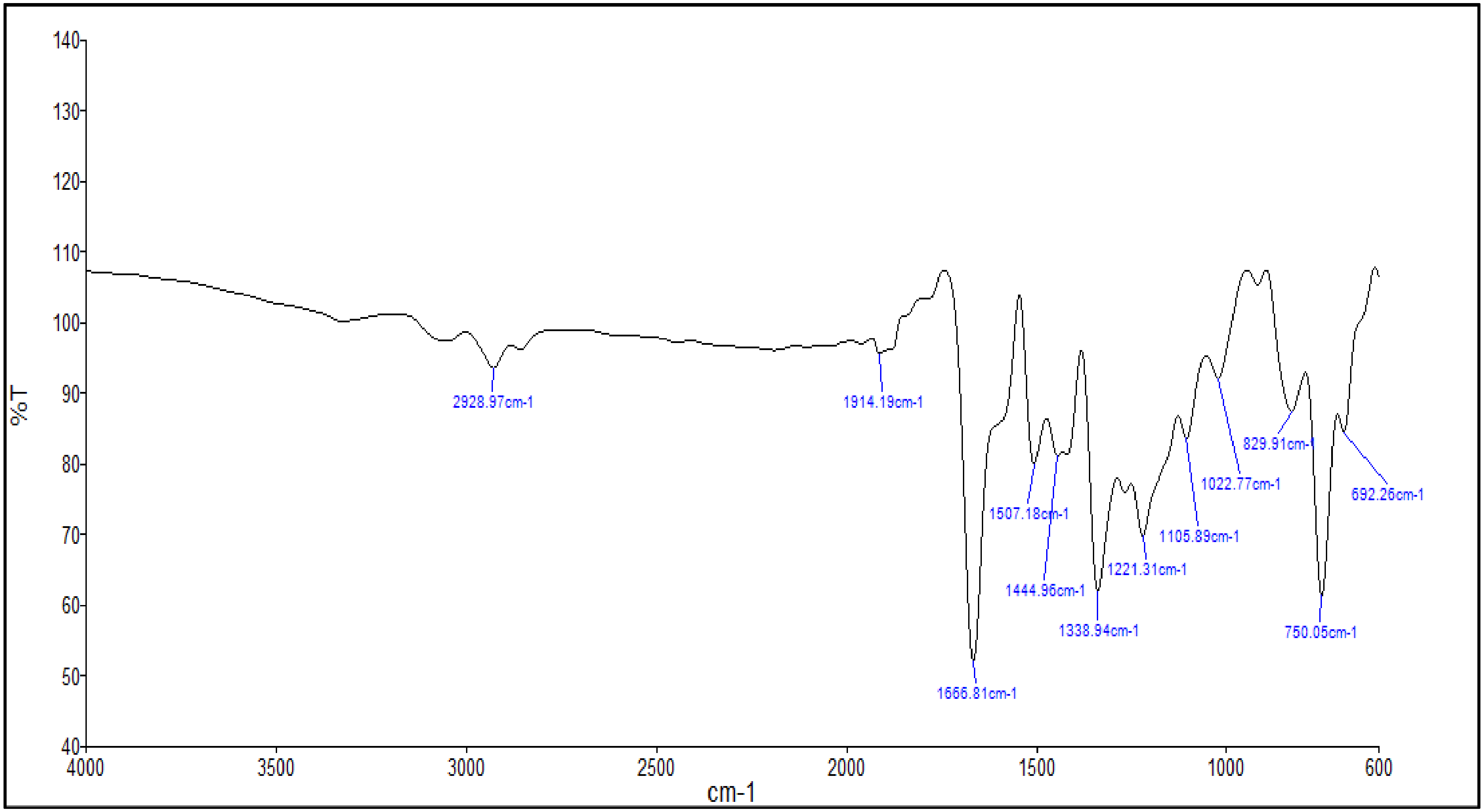

Figure S4. FTIR Spectrum for compound **4b**

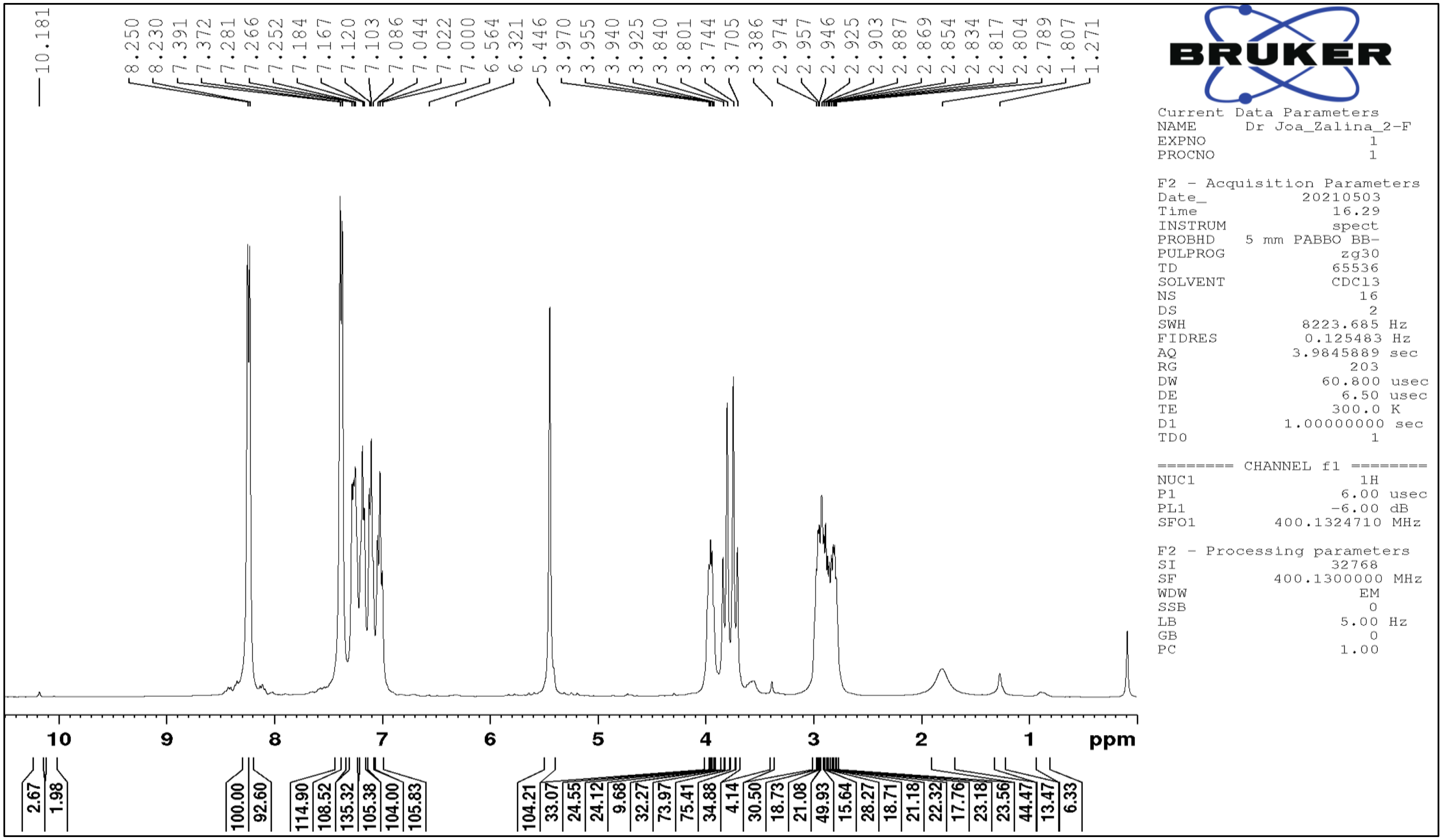

Figure S5. ^{1}H NMR Spectrum for compound **4b**

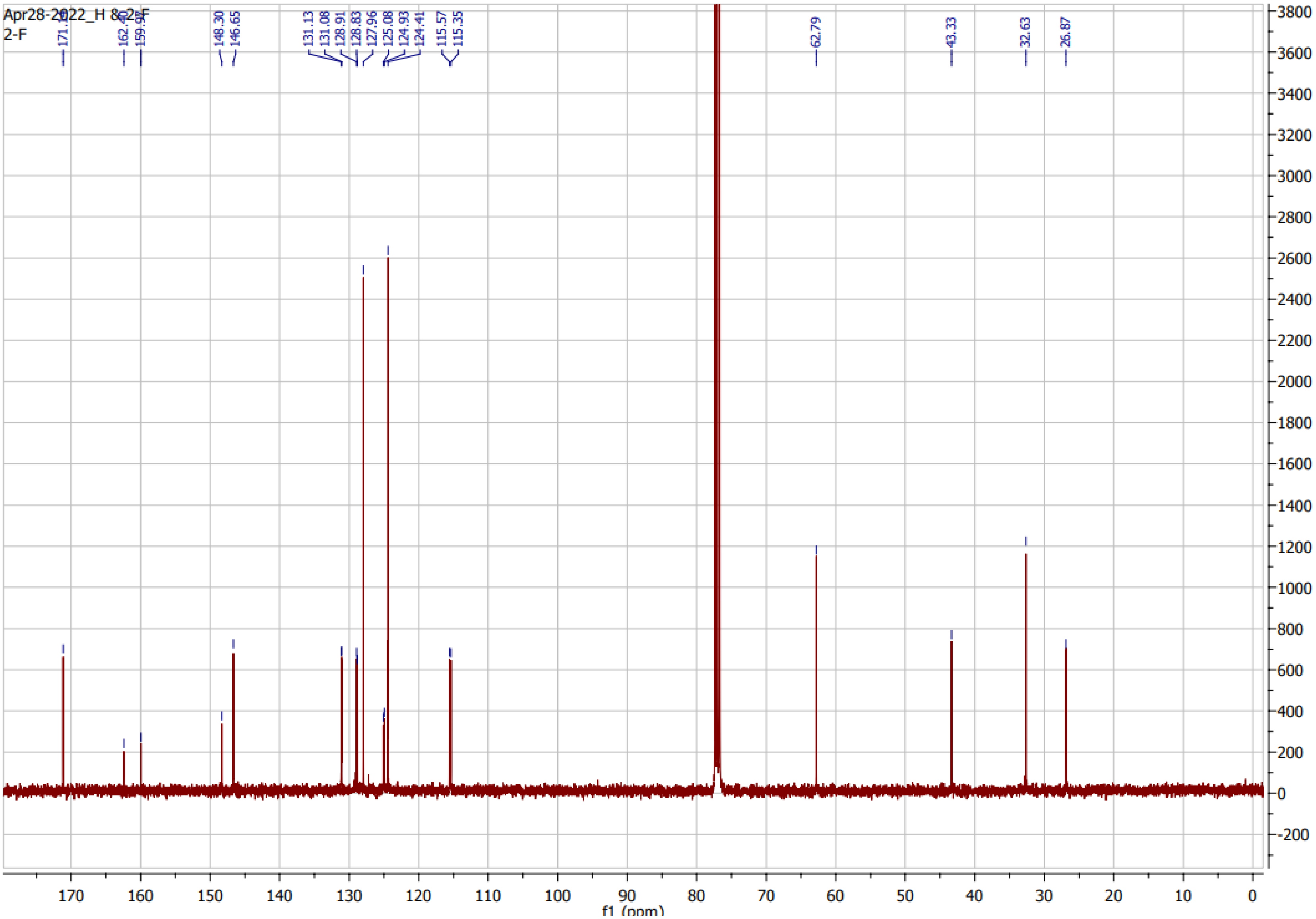

Figure S6. ^{13}C NMR Spectrum for compound **4b**

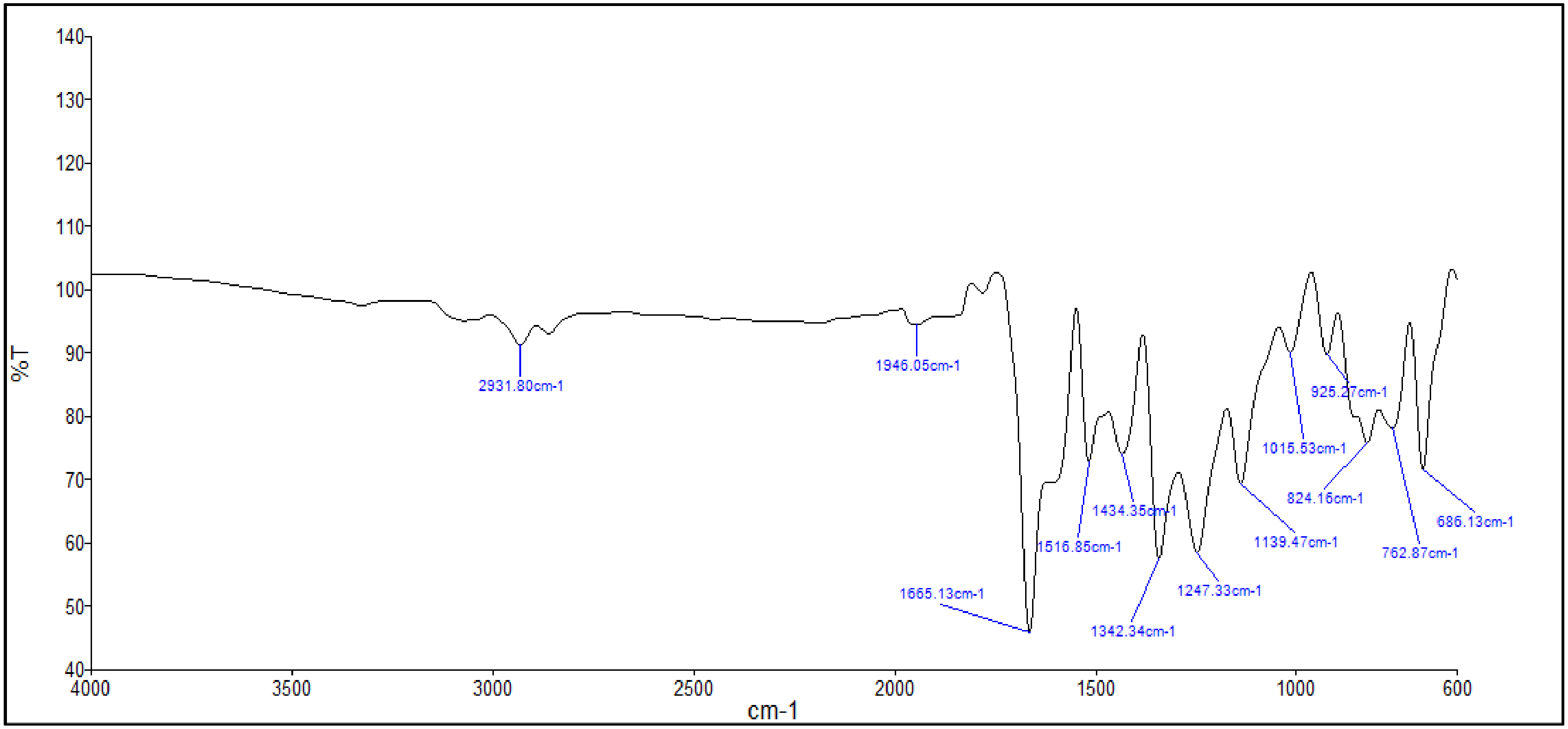

Figure S7. FTIR Spectrum for compound **4c**

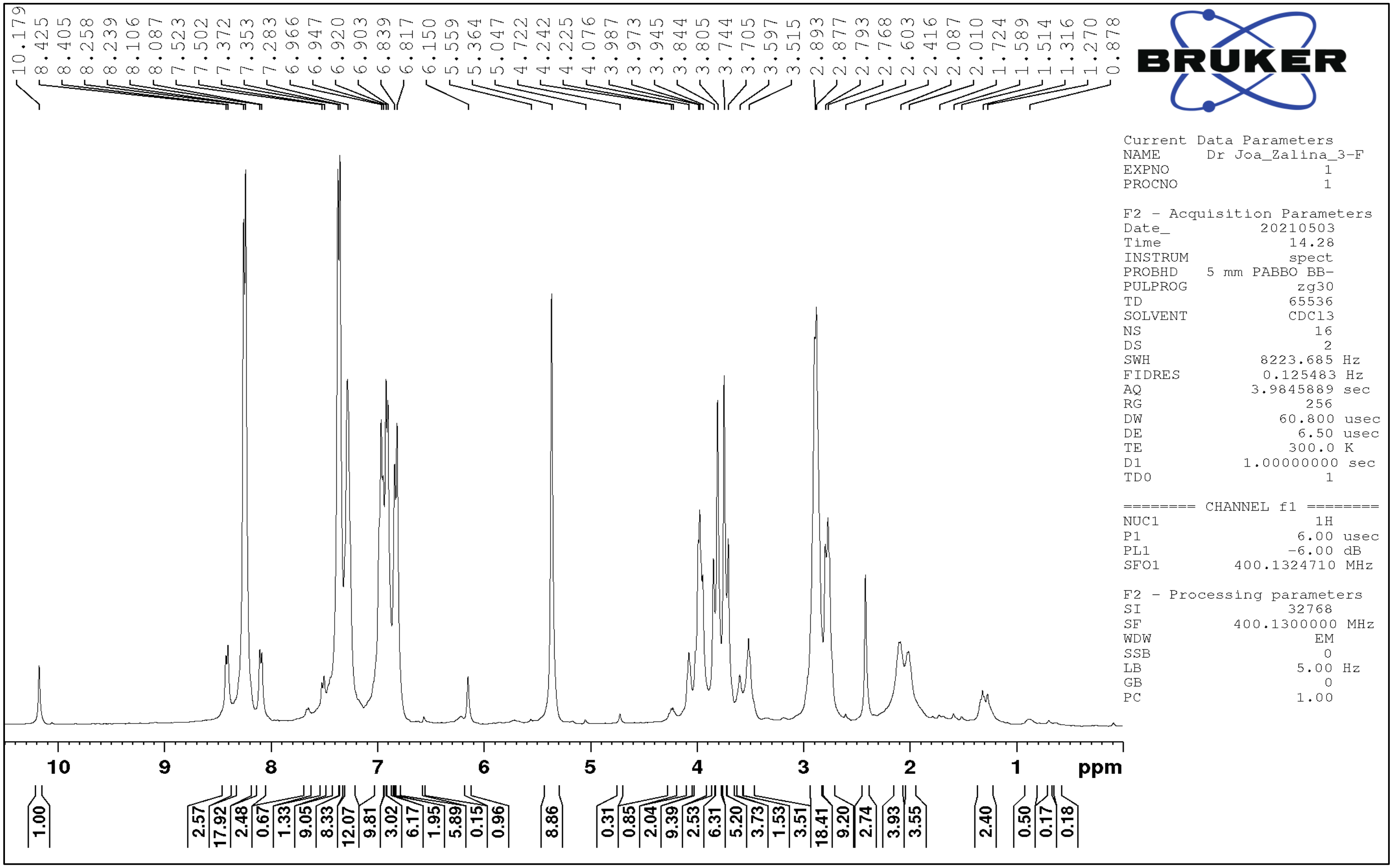

Figure S8. ^{1}H NMR Spectrum for compound **4c**

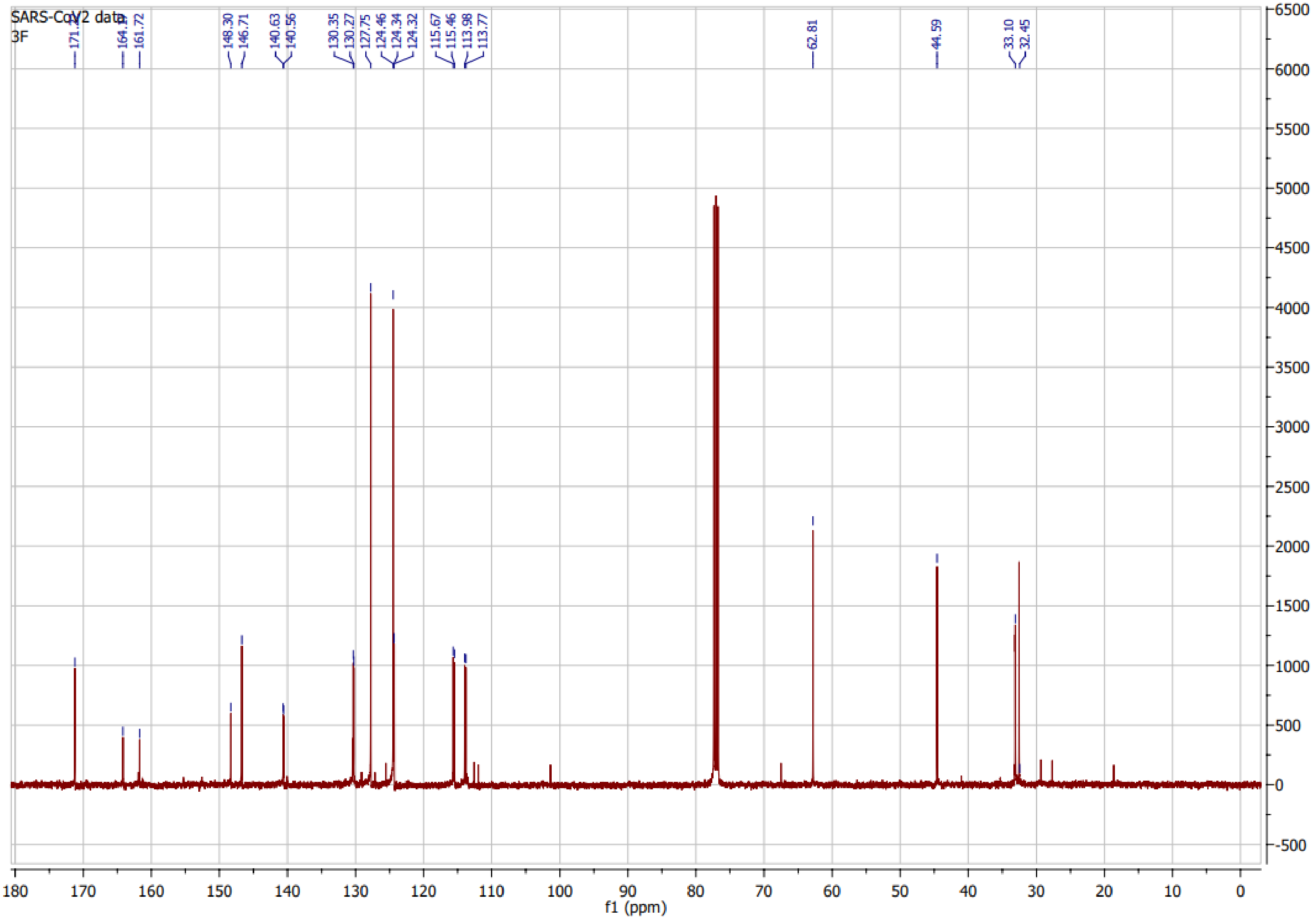

Figure S9. ^{13}C NMR Spectrum for compound **4c**

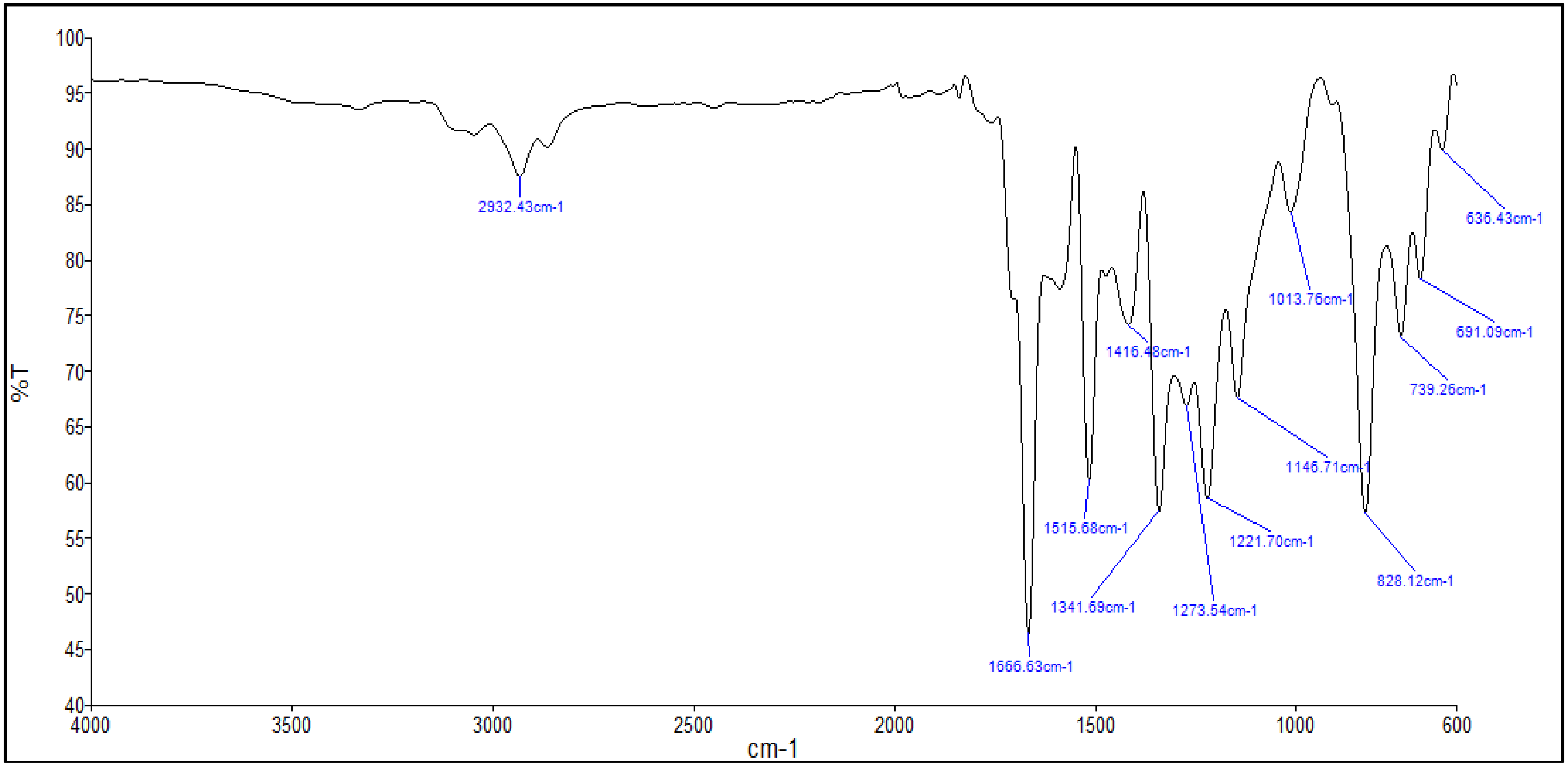

Figure S10. FTIR Spectrum for compound **4d**

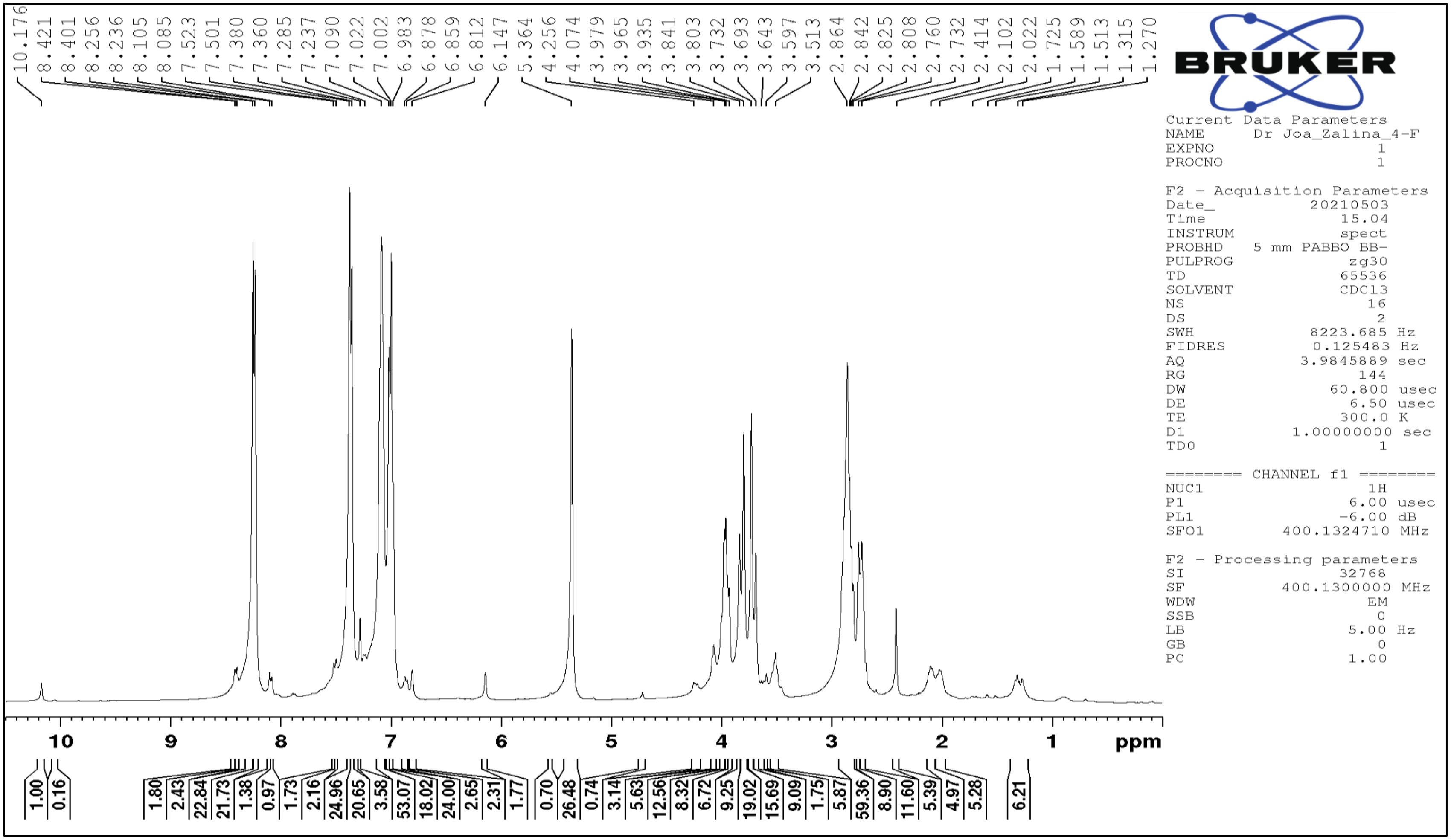

Figure S11. ¹H NMR Spectrum for compound **4d**

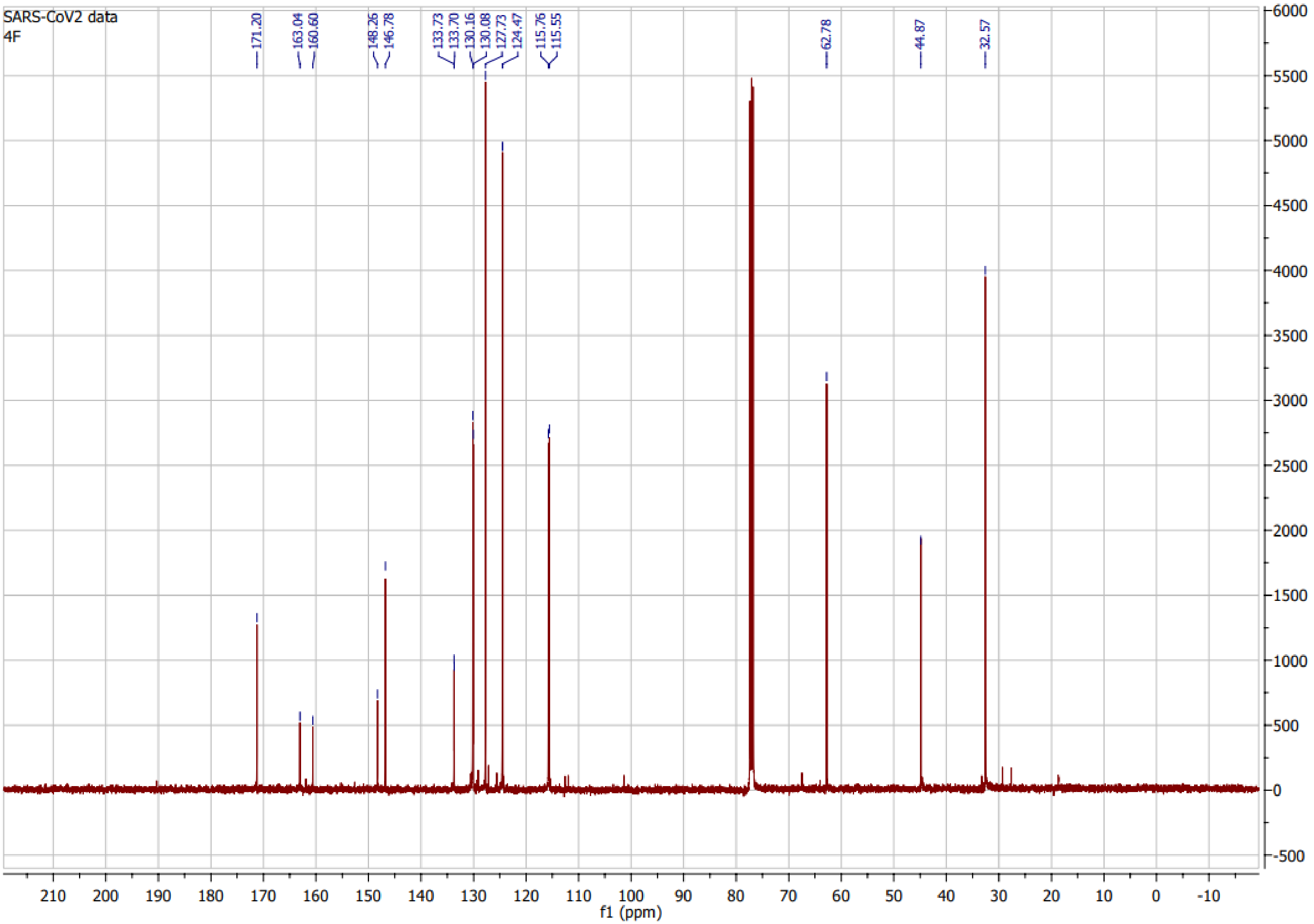

Figure S12. ^{13}C NMR Spectrum for compound **4d**

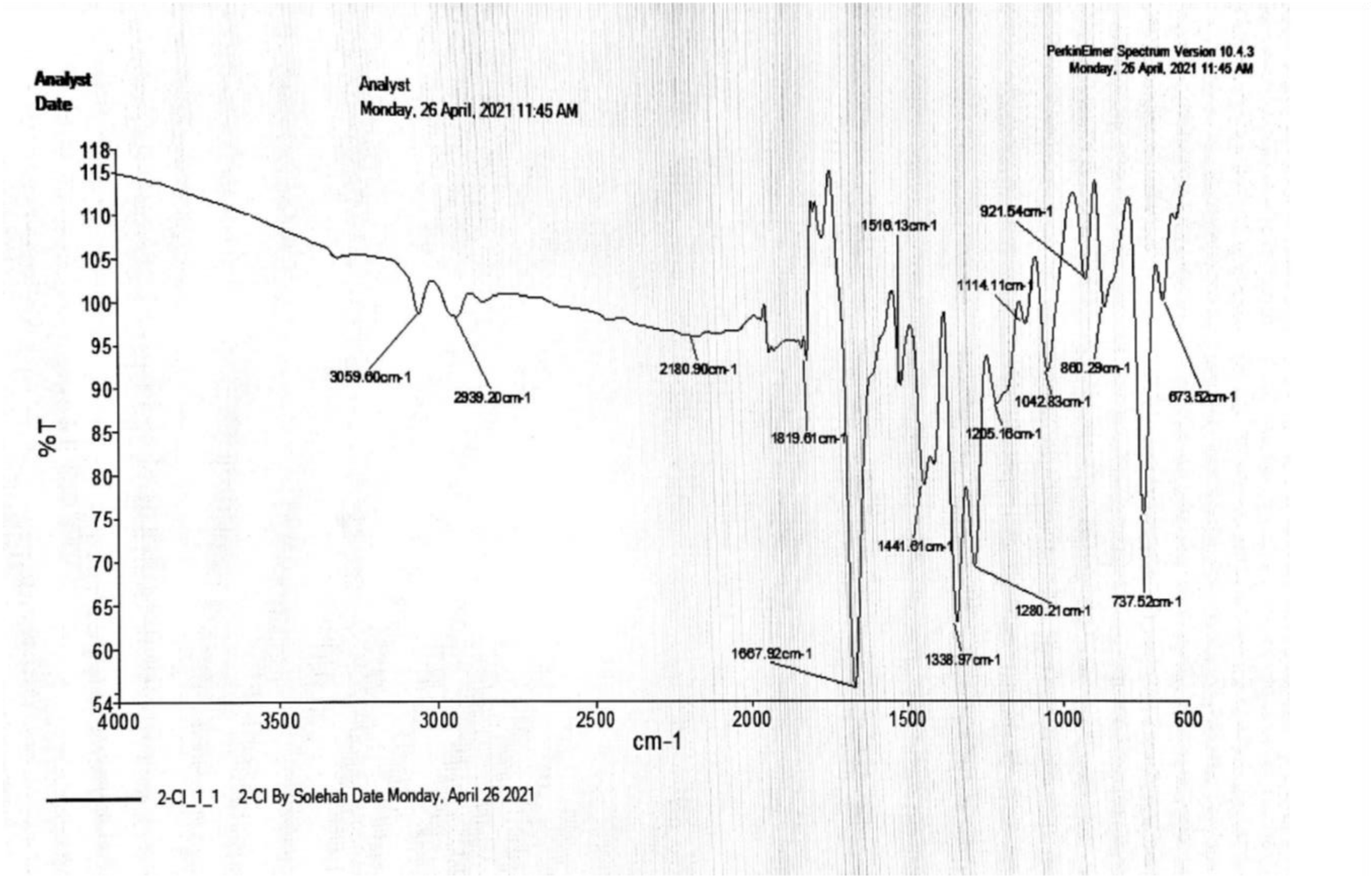

Figure S13. FTIR Spectrum for compound **4e**

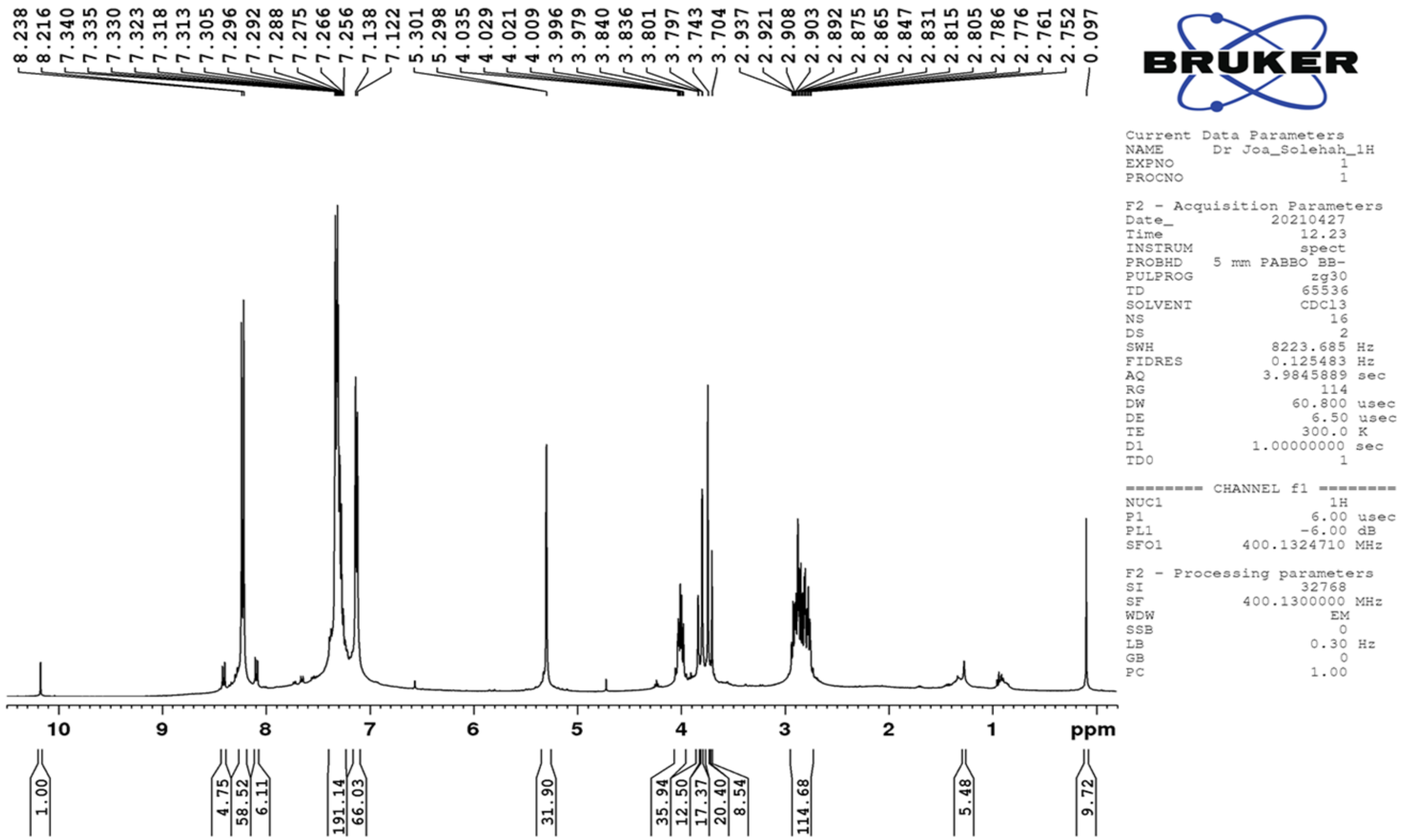

Figure S14. ^{1}H NMR Spectrum for compound **4e**

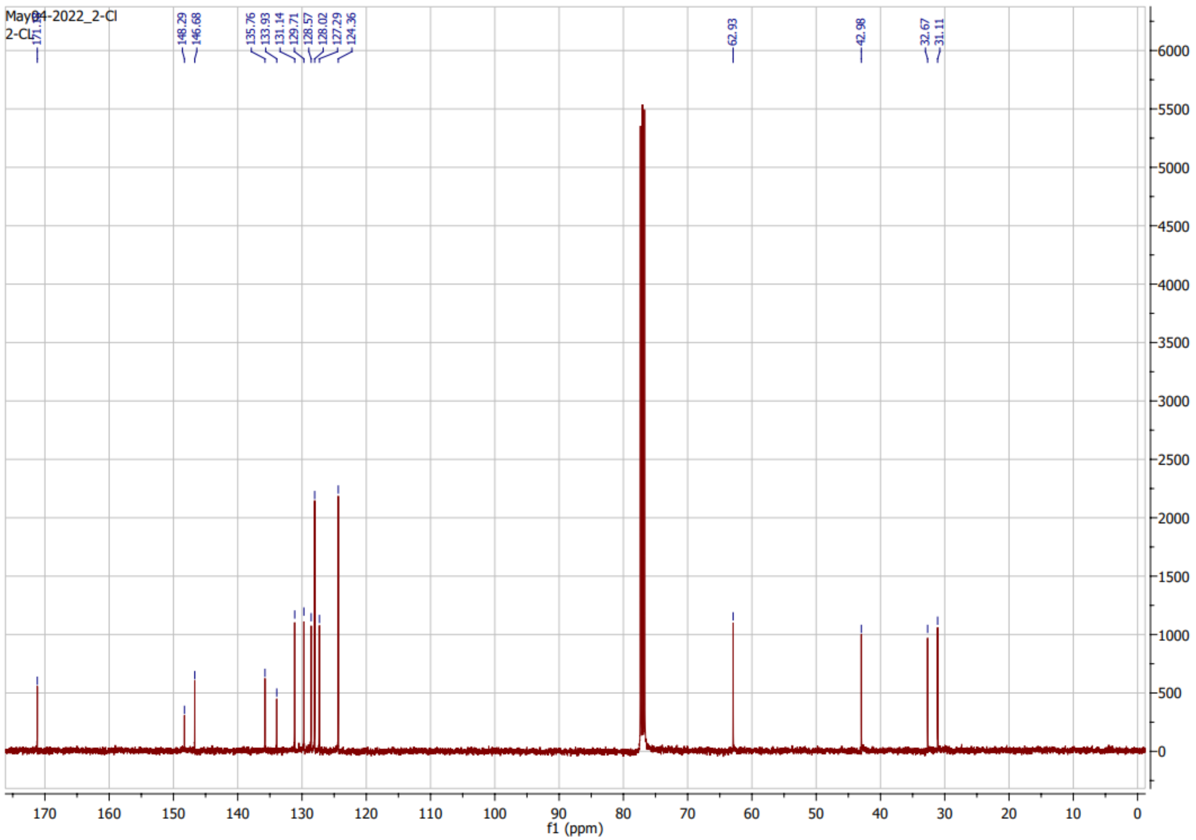

Figure S15. ^{13}C NMR Spectrum for compound **4e**

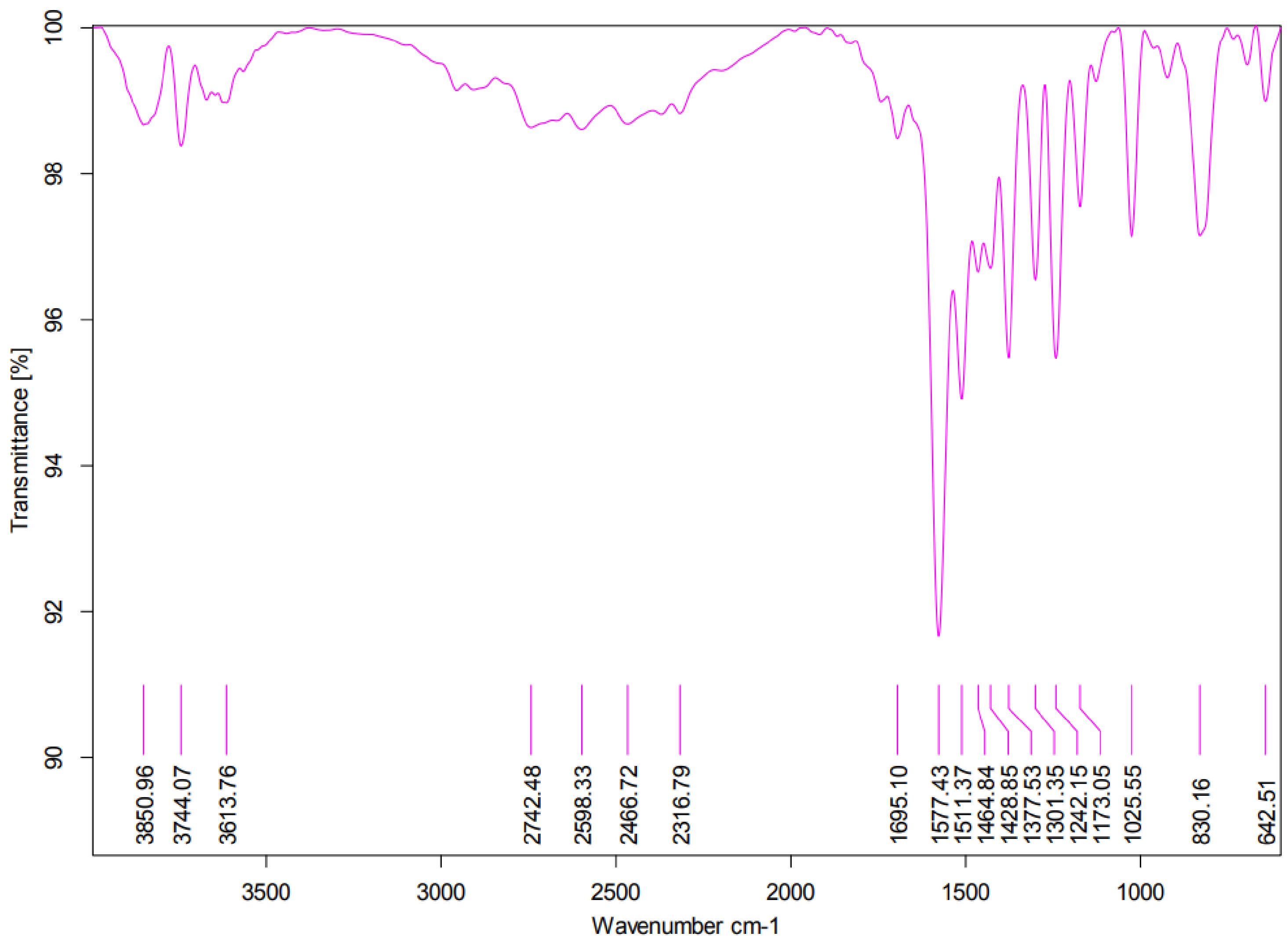

Figure S16. FTIR Spectrum for compound **4f**

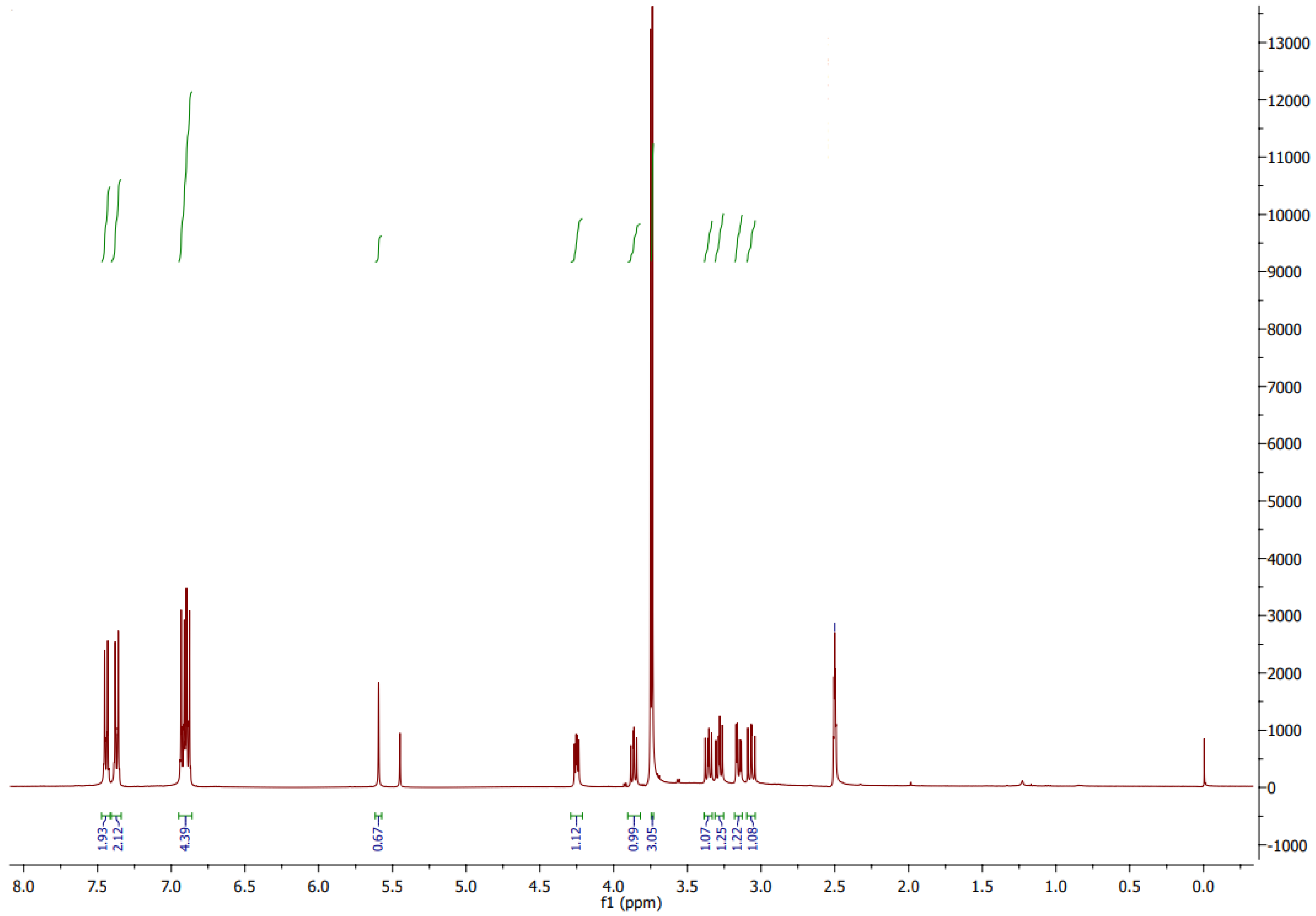

Figure S17. ^{1}H NMR Spectrum for compound **4f**

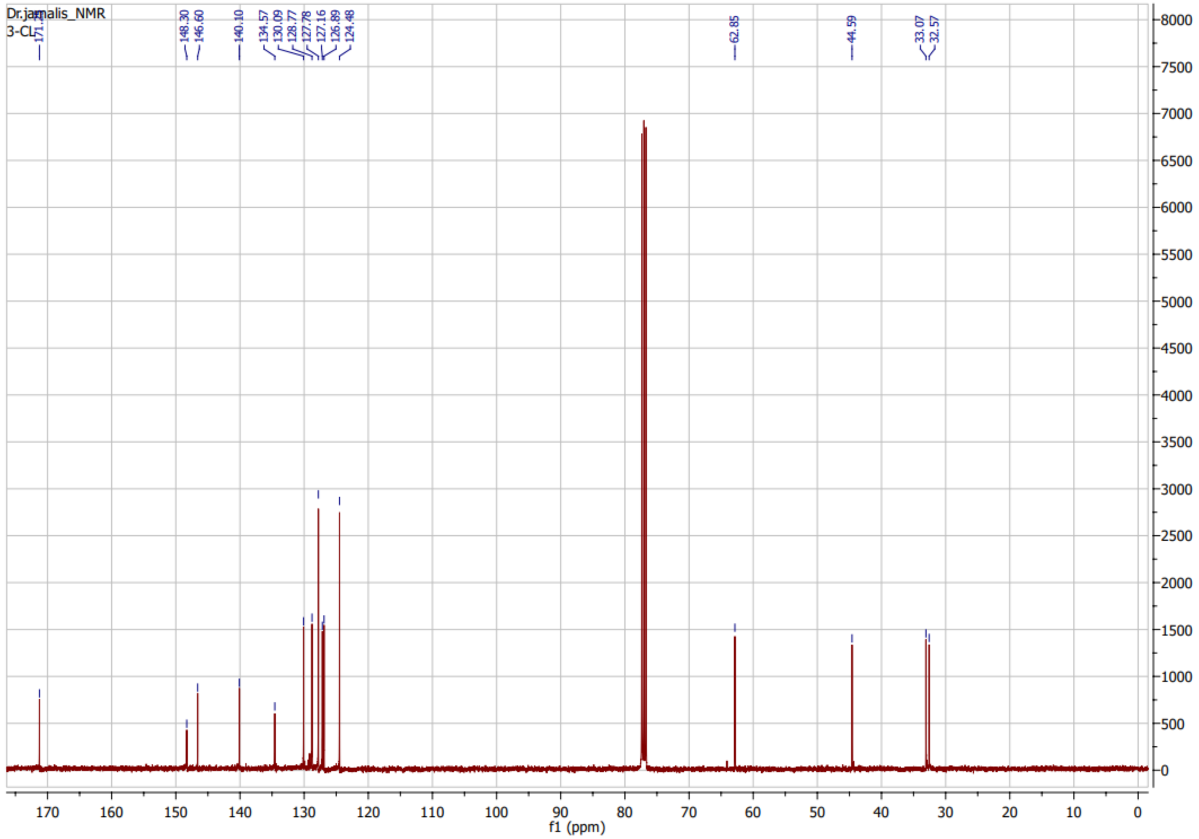

Figure S18. ¹³C NMR Spectrum for compound **4f**

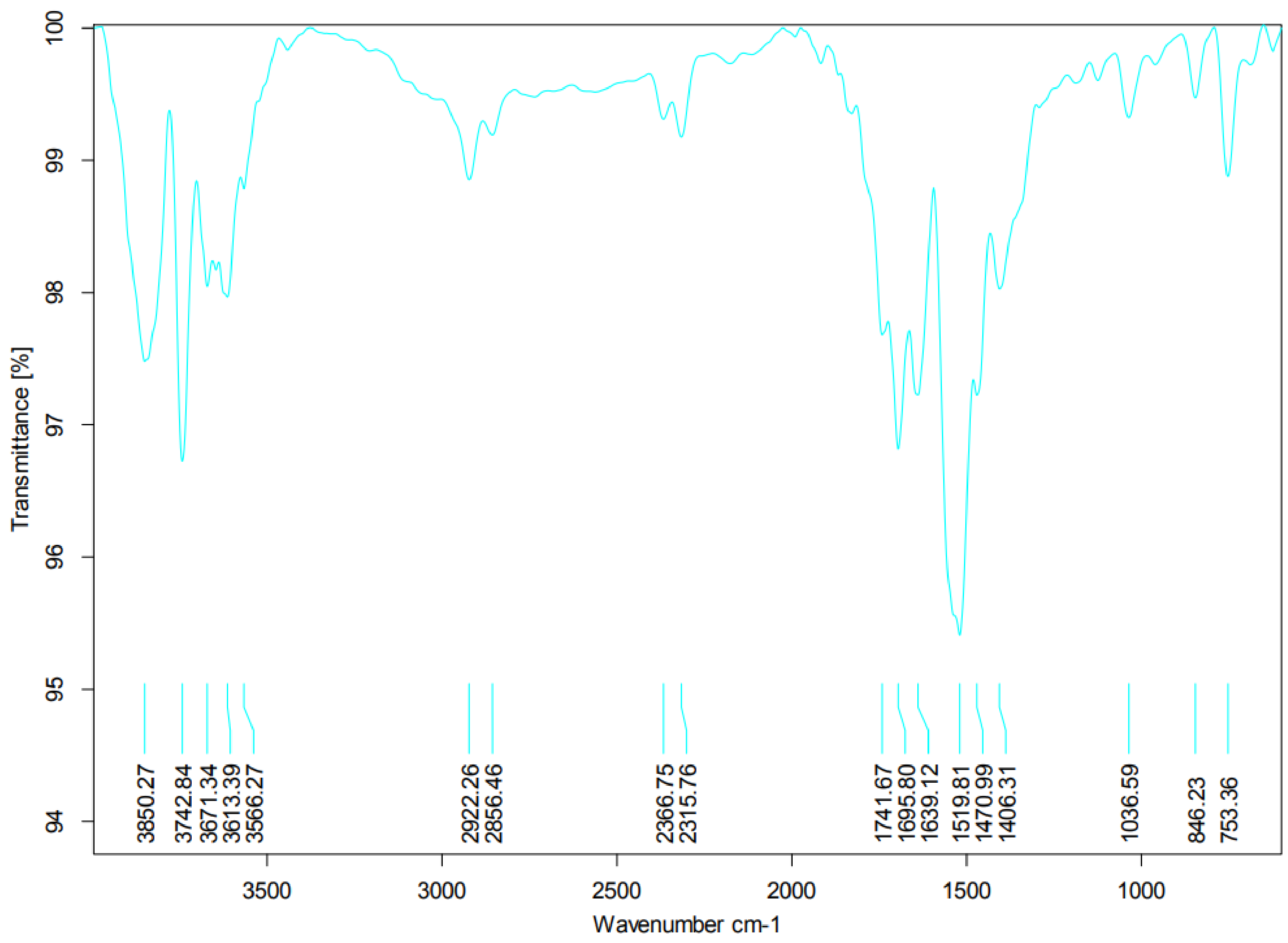

Figure S19. FTIR Spectrum for compound **4g**

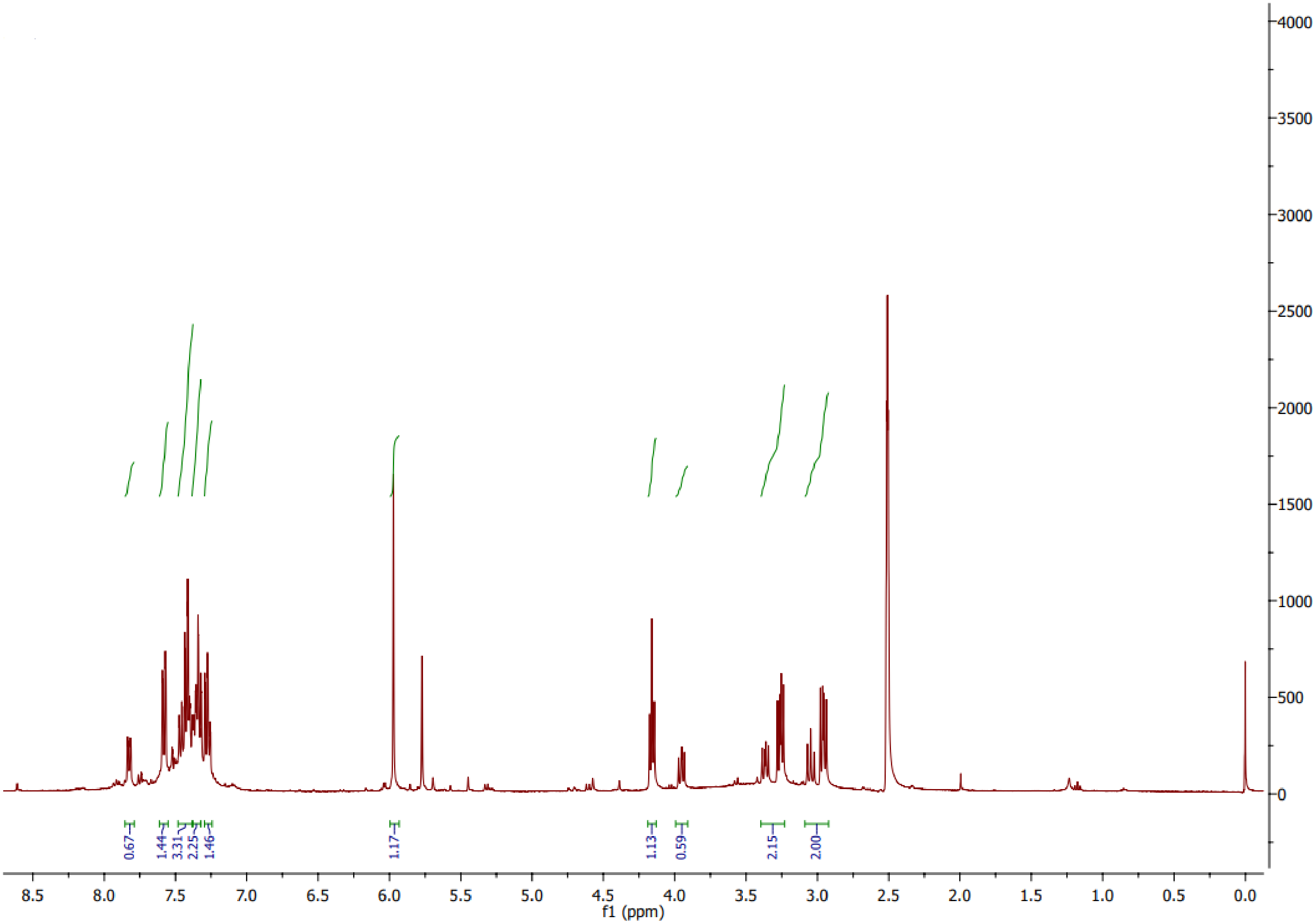

Figure S20. ^{1}H NMR Spectrum for compound **4g**

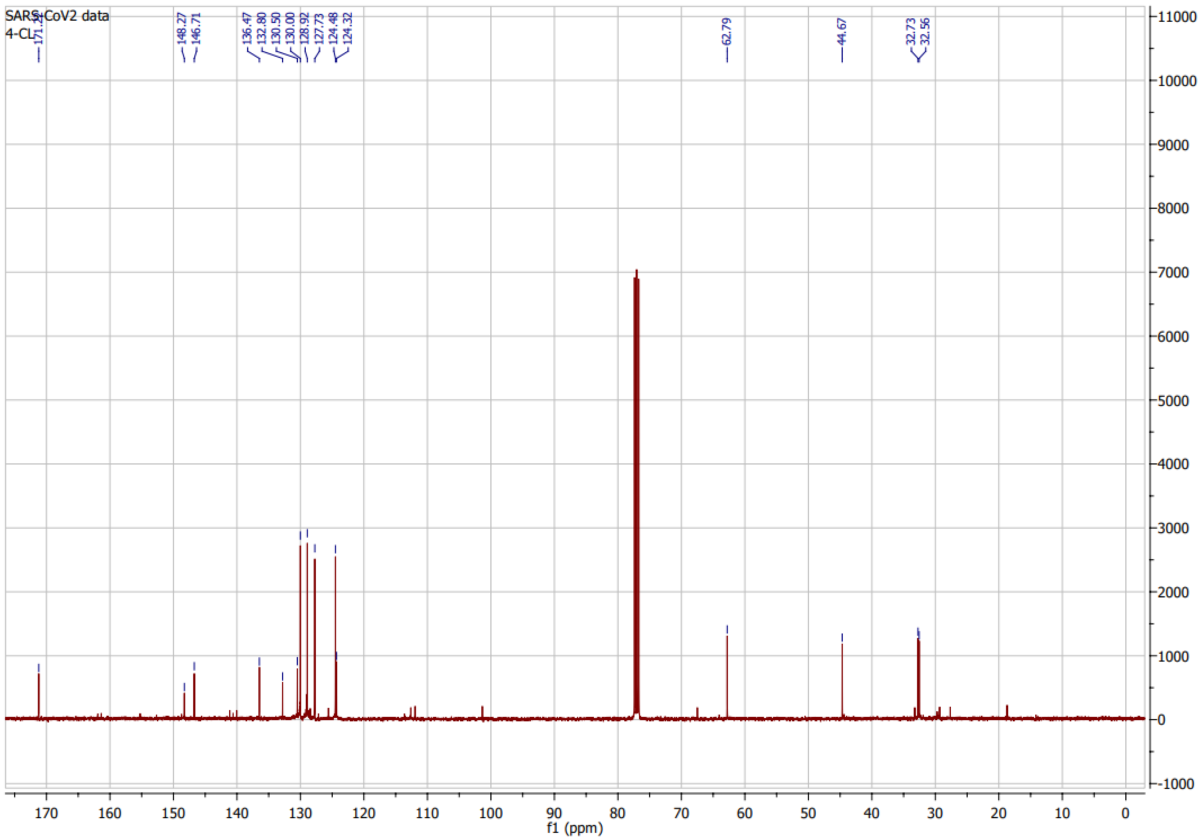

Figure S21. ^{13}C NMR Spectrum for compound **4g**

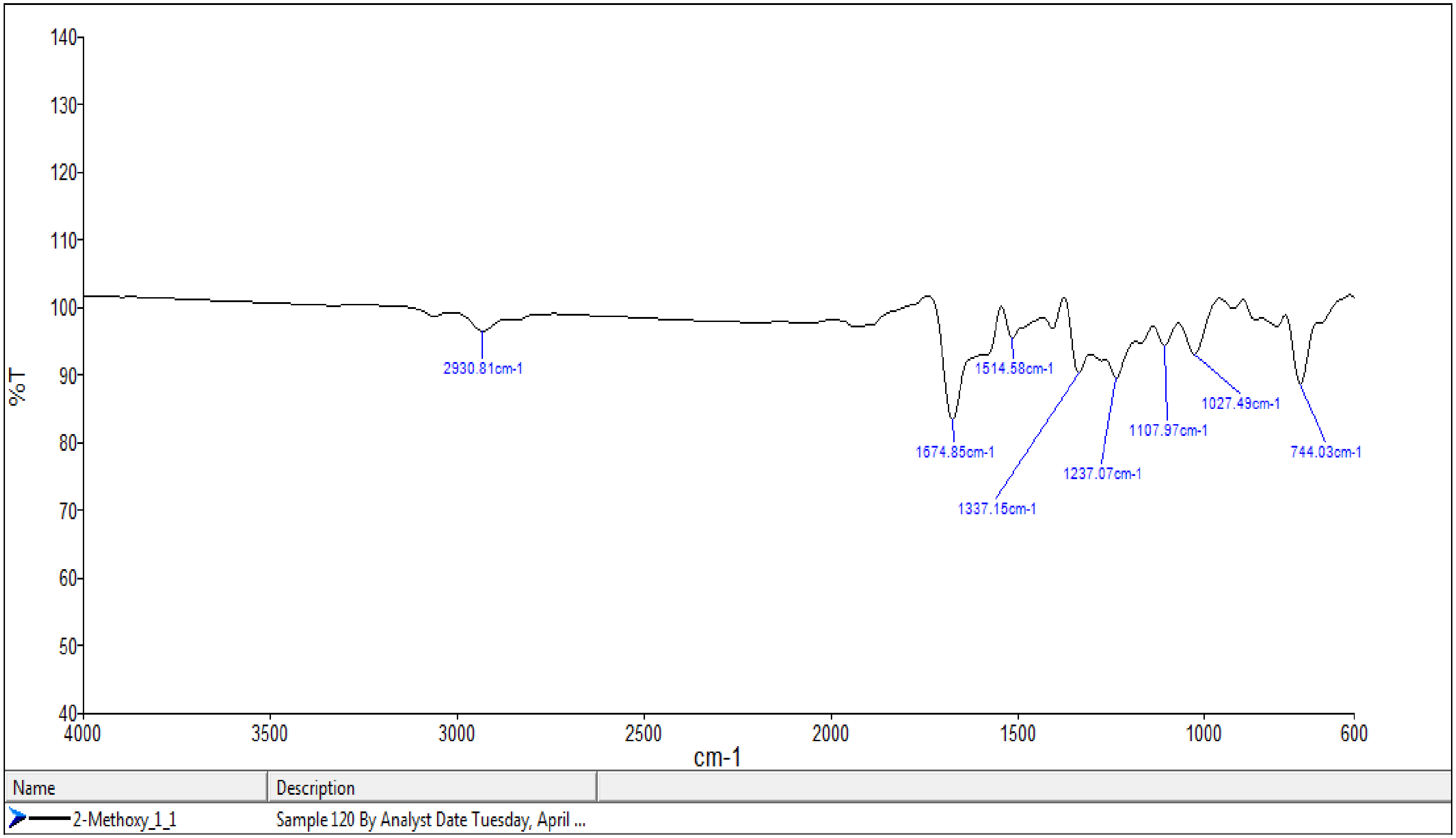

Figure S22. FTIR Spectrum for compound **4h**

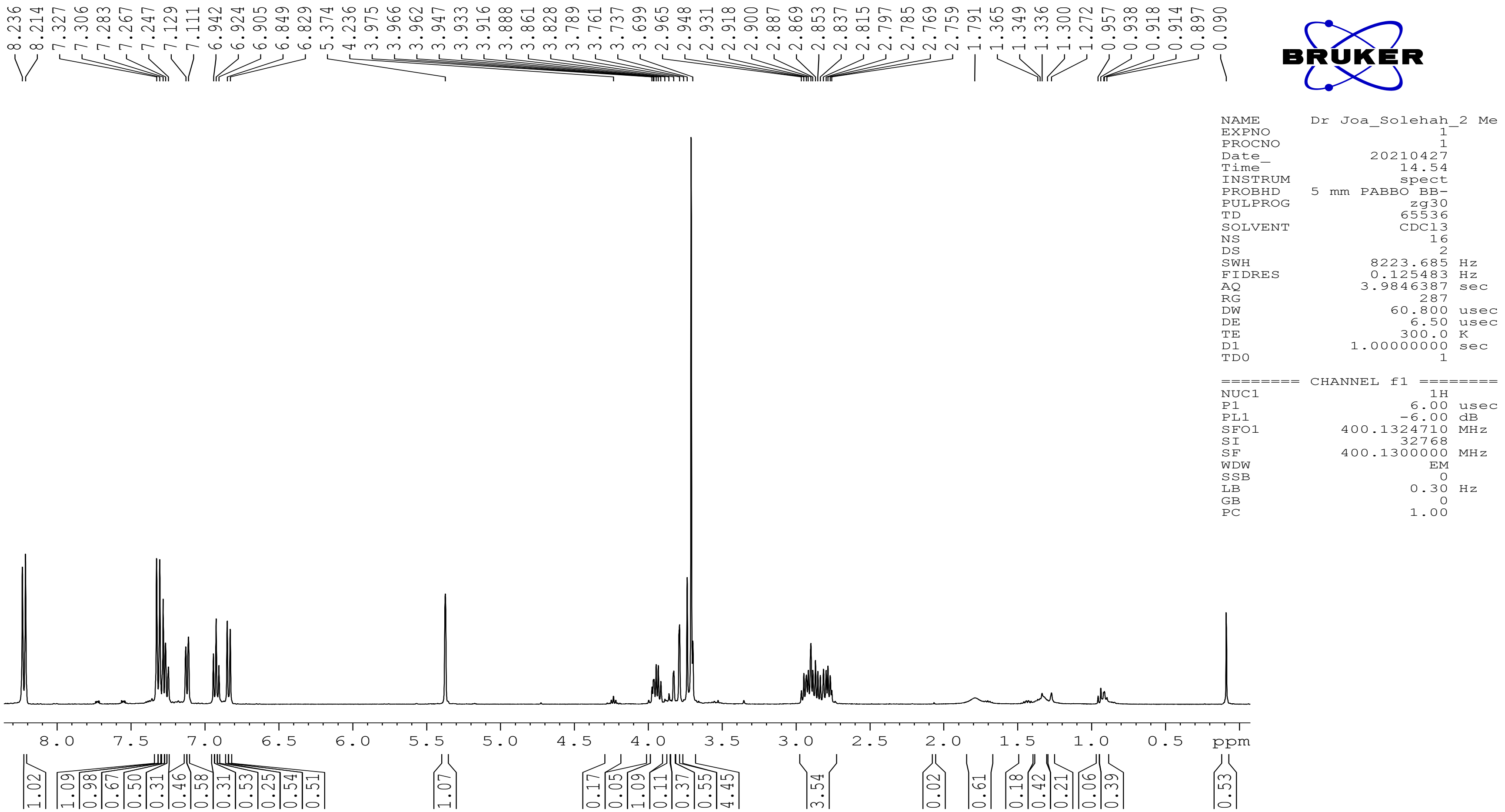

Figure S23. ^{1}H NMR Spectrum for compound **4h**

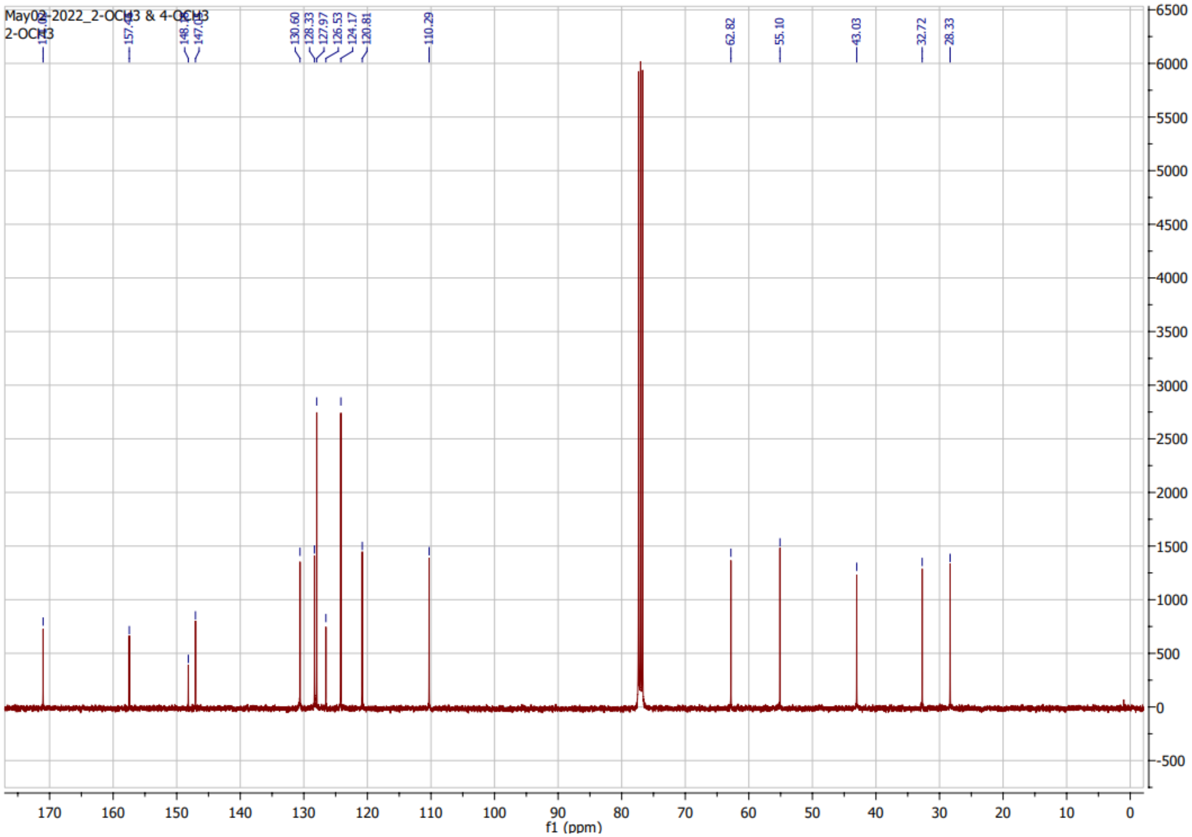

Figure S24. ^{13}C NMR Spectrum for compound **4h**

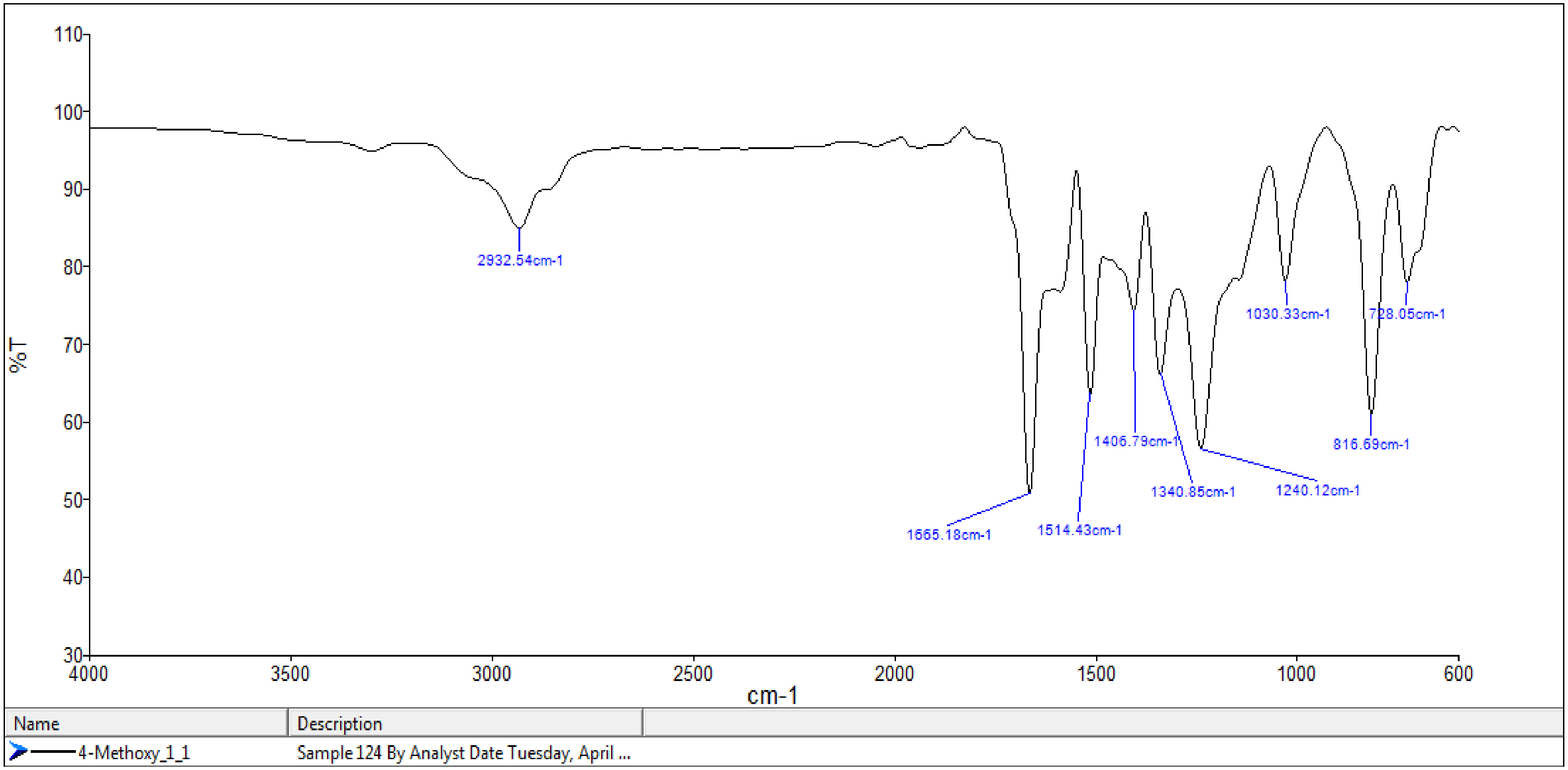

Figure S25. FTIR Spectrum for compound **4i**

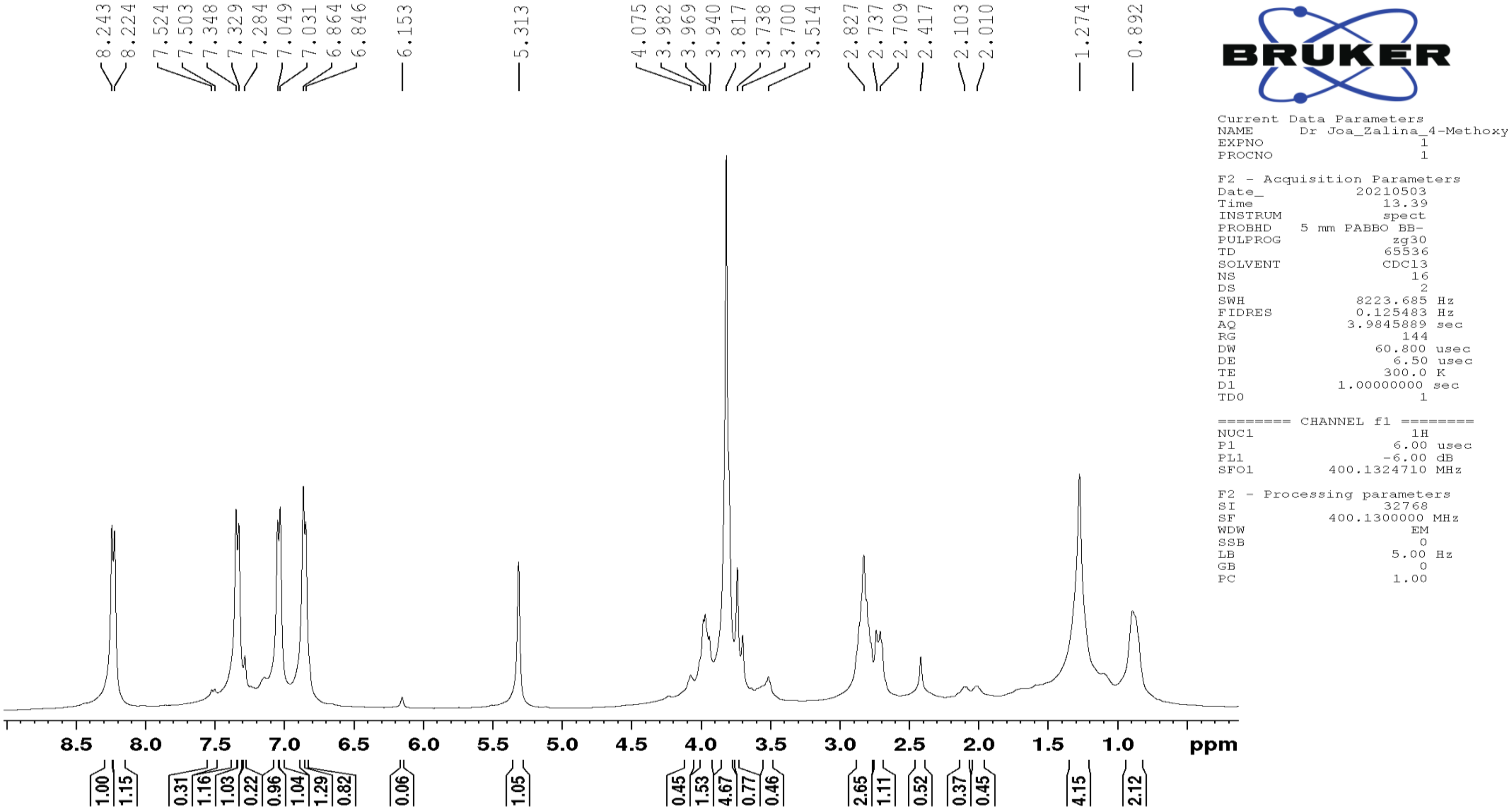

Figure S26. ^{1}H NMR Spectrum for compound **4i**

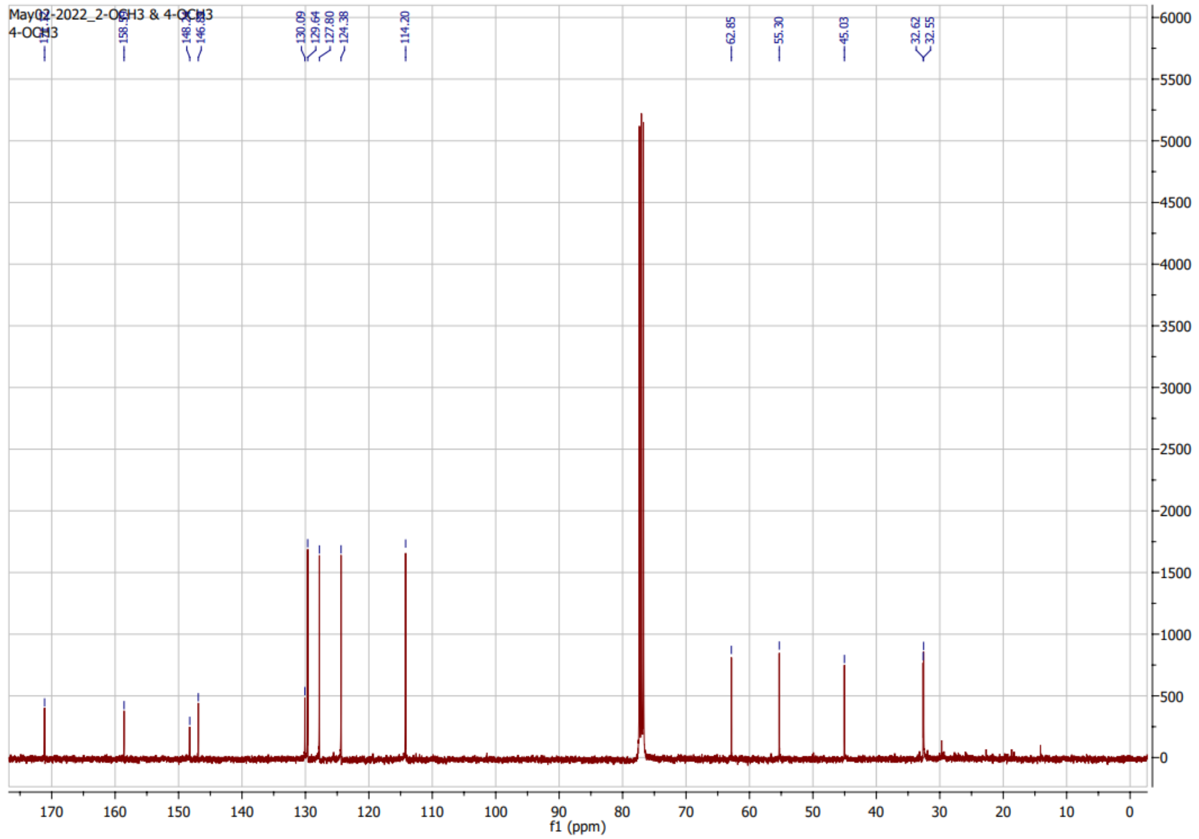

Figure S27 ^{13}C NMR Spectrum for compound **4i**
